# Regulation of cell dynamics by rapid integrin transport through the biosynthetic pathway

**DOI:** 10.1083/jcb.202508155

**Published:** 2025-12-02

**Authors:** Martina Lerche, Mathilde Mathieu, Hellyeh Hamidi, Megan Chastney, Guillaume Jacquemet, Bart Marlon Herwig Bruininks, Shreyas Kaptan, Lene Malerød, Nina Marie Pedersen, Andreas Brech, Nobuyuki Matoba, Yuichiro Sato, Ilpo Vattulainen, Pere Roca-Cusachs, Franck Perez, Gaelle Boncompain, Stéphanie Miserey, Johanna Ivaska

**Affiliations:** 1 Turku Bioscience Centre, University of Turku and Åbo Akademi University, Turku, Finland; 2 Faculty of Science and Engineering, Cell Biology, Åbo Akademi University, Turku, Finland; 3 InFLAMES Research Flagship Center, University of Turku and Åbo Akademi University, Turku, Finland; 4 Department of Physics, University of Helsinki, Helsinki, Finland; 5 Department of Molecular Cell Biology, Institute for Research, Oslo University Hospital, Montebello, Norway; 6 Centre for Cancer Cell Reprogramming, Institute of Clinical Medicine, Faculty of Medicine, University of Oslo, Montebello, Norway; 7 Department of Pharmacology and Toxicology, Brown Cancer Center, and Center for Predictive Medicine, University of Louisville School of Medicine, Louisville, KY, USA; 8 Department of Medical Pharmacy, Faculty of Pharmacy, Yasuda Women’s University, Hiroshima, Japan; 9 Institute for Bioengineering of Catalonia (IBEC), The Barcelona Institute of Technology (BIST), Barcelona, Spain; 10 Universitat de Barcelona, Barcelona, Spain; 11 Intracellular Transport: Engineering and Mechanisms Laboratory, PSL Research University, Sorbonne Université, Centre National de la Recherche Scientifique, Institut Curie, Paris, France; 12 Department of Life Technologies, University of Turku, Turku, Finland; 13 Western Finnish Cancer Center (FICAN West), University of Turku, Turku, Finland; 14 Foundation for the Finnish Cancer Institute, Helsinki, Finland

## Abstract

Constitutive integrin endocytosis and recycling control cell movement and morphology. In contrast, the role of newly synthesized integrins delivered via the biosynthetic pathway has been largely overlooked. We used the retention using selective hooks system to monitor the localization of new integrins exiting the endoplasmic reticulum in space and time. We discovered that new integrin delivery to the plasma membrane is polarized and enhances cell protrusion and focal adhesion growth in an extracellular matrix-ligand–dependent manner. Motor-clutch modeling explained the increased adhesion as higher integrin availability driving recruitment of additional receptors. Unexpectedly, live-cell imaging revealed a small subset of fast-emerging integrin vesicles rapidly transported to the cell surface to facilitate localized spreading. This unconventional secretion depended on cell adhesion and correlated with increased surface levels of immature, high-mannose glycosylated integrin, indicating bypass of the canonical Golgi-dependent secretory pathway. Thus, spatial plasma membrane-targeting of new integrins rapidly alters adhesion receptor availability, providing cells with added plasticity to respond to their environment.

## Teaser

The study reveals that newly synthesized integrins can bypass the Golgi to be actively delivered to specific plasma membrane regions, where they enhance cell protrusion and adhesion. This targeted secretion rapidly increases integrin availability, giving cells dynamic flexibility to adjust to their environment.

## Introduction

Cells sense and respond to the extracellular matrix (ECM) through integrins and integrin localization at the plasma membrane is essential for focal adhesion (FA) formation. Numerous studies have addressed the dynamics of FA assembly as a function of integrin diffusion along the plasma membrane, and integrin activation and tethering to the cytoskeleton through components of the integrin adhesion complexes, such as talin and vinculin (Paszek et al., 2014; Saltel et al., 2009; Humphries et al., 2007). On the other hand, FA disassembly has been investigated from the point of view of proteolytic cleavage of adhesion components, microtubule-dependent adhesion turnover and integrin endocytosis from the plasma membrane (Yue et al., 2014; Ezratty et al., 2009; Chan et al., 2010). In addition, the recycling of plasma membrane endocytosed integrin back to the cell surface has been extensively studied and implicated in the generation of local cell protrusions, co-trafficking with growth-factor receptors, cell invasion and system-level metastasis (Astro et al., 2016; Caswell et al., 2008; Alanko et al., 2015). These studies, however, have focused on endo/exocytic traffic of mature plasma membrane integrins. In contrast, very little is known about the targeting of newly synthesized integrins to the plasma membrane and their contribution to cell adhesion, spreading and FA dynamics. The relevance of integrin biosynthetic traffic is virtually unexplored and overlooked in the regulation of cell dynamics, predominantly owing to a lack of suitable methodology.

Integrins are a family of 24 heterodimeric cell-surface receptors composed of a larger α-subunit and a smaller β-subunit. The β1-subunit constitutes 12 integrin heterodimers, which mediate adhesion to a variety of ECM molecules (Chastney et al., 2020). Only relatively few studies have investigated integrin maturation and delivery to the plasma membrane. Early investigations exploring the regulation of integrin expression with pulse-chase metabolic labeling have shown that the rate of integrin heterodimer assembly and maturation is determined by the availability of integrin α-subunits (Heino et al., 1989). The β1-subunits are produced in an excess ratio and are retained in their immature form in the endoplasmic reticulum (ER) where they await to assemble with newly synthesized α-integrins (Heino et al., 1989; De Strooper et al., 1991; Lenter and Vestweber, 1994). In the ER, newly synthesized integrins bind to Ca^2+^, which maintains receptors in an inactive bent conformation until they reach the cell surface. Ca^2+^ depletion blocks the trafficking of α5β1-integrins from the ER to the Golgi (Tiwari et al., 2011). Talin plays an important role in trafficking of newly synthesized integrins, as talin depletion causes accumulation of α5β1-integrins in the early secretory pathway (Martel et al., 2000). How talin regulates exocytosis of newly synthesized integrins remains unknown, and other players in the regulation of integrin biosynthetic traffic are yet to be identified. To date, studies on newly synthesized integrins have been based on metabolic pulse-chase labeling and utilization of conformation-specific antibodies, which have limitations for real-time studies and visualization of integrin delivery. The development of the retention using selective hooks (RUSH) assay has permitted real-time tracking of newly synthesized proteins (Boncompain et al., 2012). A recent study employing this method concluded that post-Golgi carriers are not transported randomly to the cell surface. Instead exocytosis of versatile cargo is focused to areas close to FAs (Fourriere et al., 2019). However, whether this holds true for integrins and whether their delivery is influenced by specific ECM composition or ECM rigidity remains unknown.

Here, we have generated a fully functional, extracellularly tagged α5-integrin subunit and coupled it to the RUSH system to explore context-dependent traffic of newly synthesized fibronectin (FN)-binding α5β1-integrin to the plasma membrane. We demonstrate that (1) delivery of newly synthesized integrins to the plasma membrane is faster than previously thought; (2) their polarized delivery and ECM ligand–dependent recruitment to FAs facilitate dynamic cell responses to ECM cues providing an additional, thus far unrecognized level of integrin regulation; (3) a small subset of integrin α5 is delivered in a high-mannose glycosylation state to the plasma membrane via Golgi-bypass trafficking. These findings are the first demonstration of biosynthetic integrin traffic influencing FA maintenance and cell dynamics.

## Results

### Molecular simulation–guided generation of RUSH-α5

**Figure S1.**
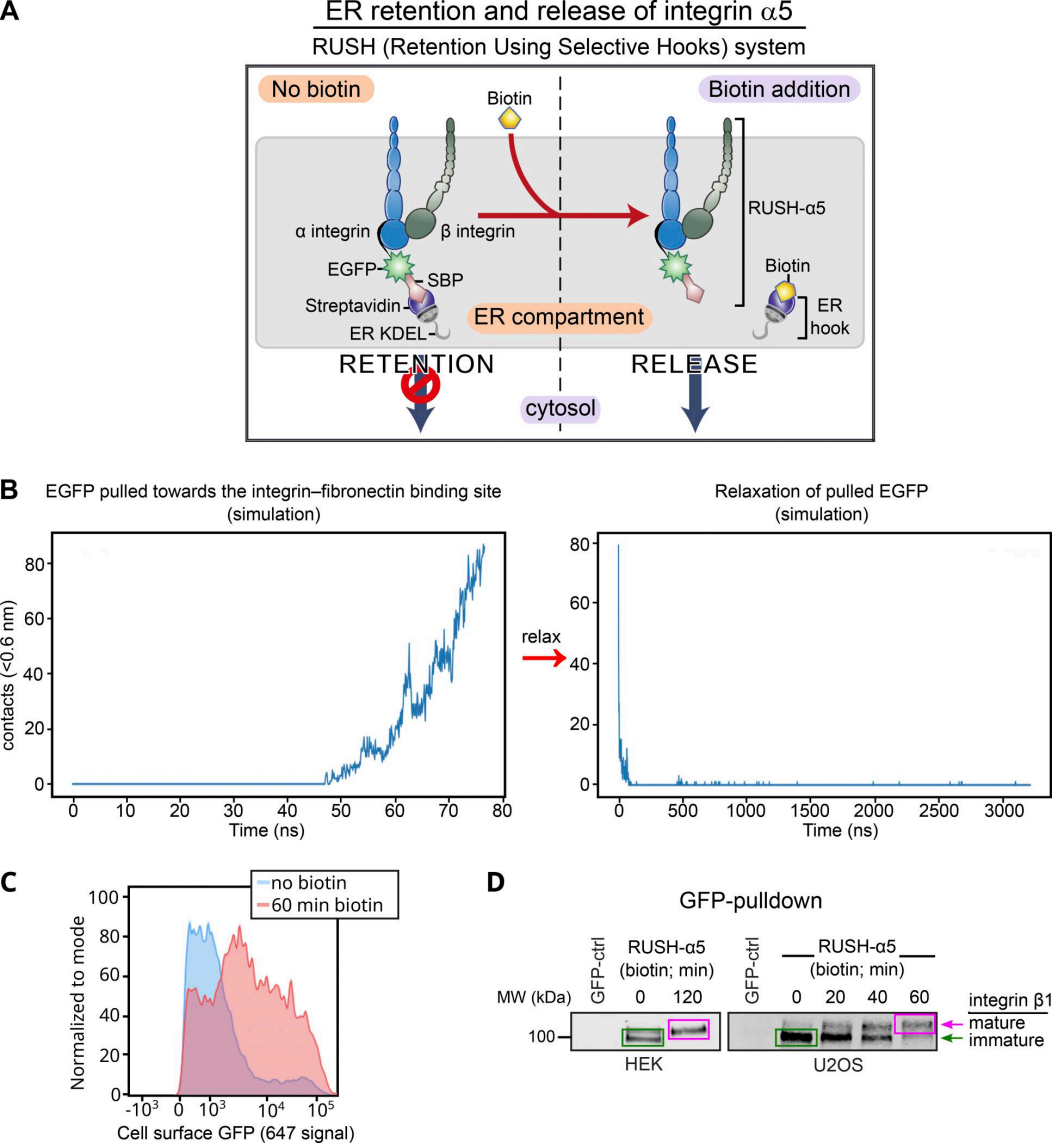
**The RUSH system applied to integrin α5. (A)** Principles of the RUSH-α5 integrin. In all experiments, SBP-EGFP-ITGA5 (RUSH-α5) is co-expressed with streptavidin-KDEL (ER-hook). In the absence of biotin, this combined complex is retained within the ER. Biotin addition displaces the ER-hook and releases RUSH-α5 into the cytoplasm. **(B)** The number of contacts between EGFP and FN during simulations of the coarse-grained model. Left: simulation of EGFP being pulled towards the FN-binding site, starting when the C-terminus of the EGFP and the N-terminus of the integrin α5 are <1 nm apart, the linker included, leading to the formation of contacts (Video 2 A). Right: simulation of a fully stretched EGFP, initially in close proximity to the FN-binding site, that is allowed to relax without a biasing force resulting in a spontaneous and rapid loss of contacts (<100 ns; Video 2 B). The pulling process spanned 8 nm and 80 ns. The relaxation spanned 3200 ns. Contacts were calculated between EGFP and FN with a cutoff of 0.6 nm. **(C and D)** RUSH-α5 is expressed on the cell surface and forms a functional heterodimer with integrin β1. **(C)** Representative flow cytometry analysis of cell surface RUSH-α5 levels (detected with the anti-GFP-AF647 antibody) in RUSH-α5–expressing U2OS cells ± biotin. **(D)** Representative immunoblots of GFP pulldowns performed in RUSH-α5 or control transfected cells ± biotin treatment for the indicated times and probed for endogenous integrin β1. The faster migrating band of immature integrin β1 is indicated by a green arrow and box and the slower migrating band of mature integrin β1 with a magenta arrow and box. Source data are available for this figure: SourceData FS1.

**Figure 1.**
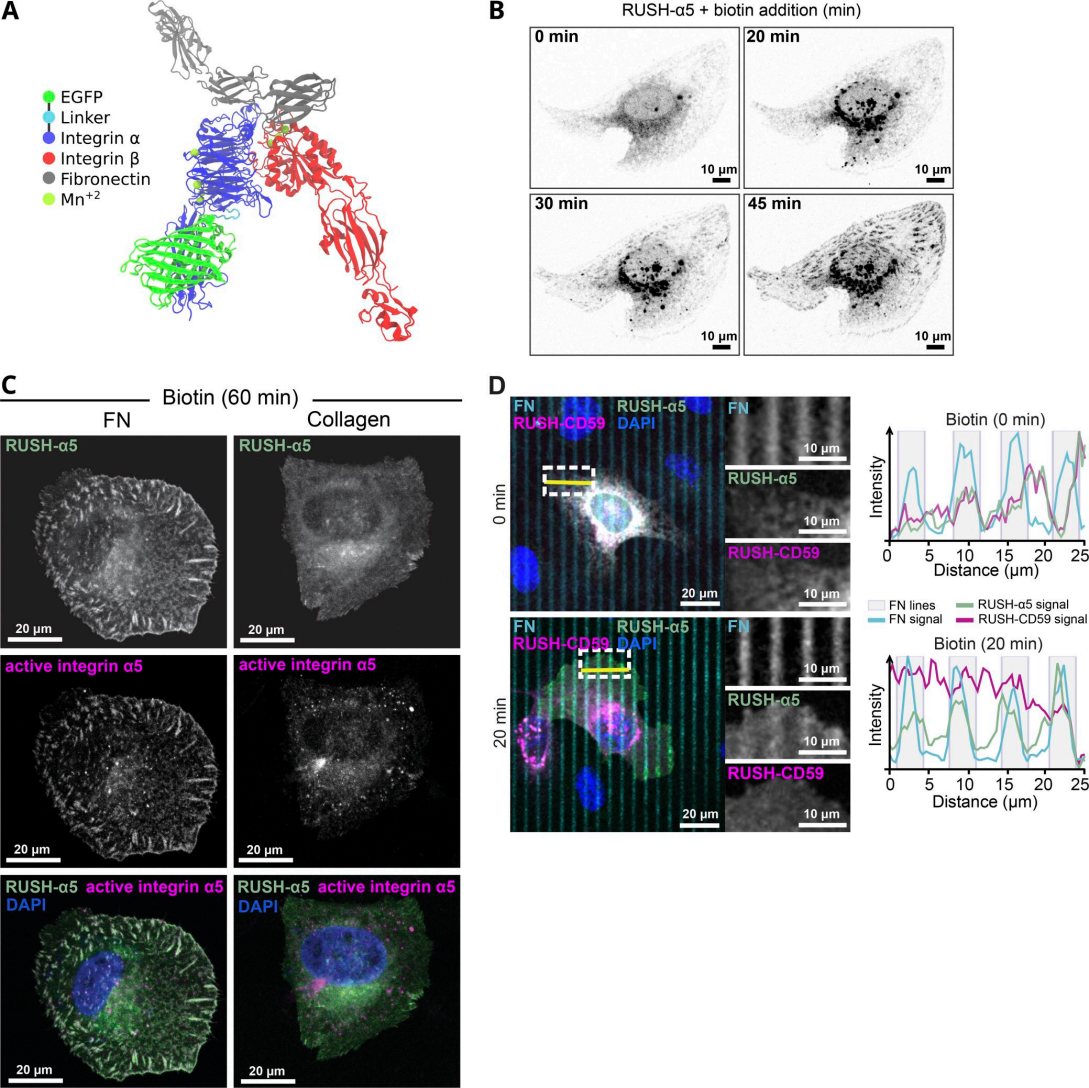
**RUSH-α5 delivery to the plasma membrane is spatially regulated by the ECM. (A)** Model of RUSH-α5 (EGFP-integrin α5)–integrin-β1 heterodimer based on the structure of human α5β1-integrin bound to FN (PDB: 7NWL) (see also Video 1). **(B)** Representative immunofluorescence timelapse images of a U2OS cell expressing RUSH-α5 (SBP-EGFP-integrin α5) and plated on FN ± biotin treatment (single grayscale images for the indicated time points are shown; see Video 4). **(C)** Representative immunofluorescence images of RUSH-α5 and active integrin α5β1 (SNAKA51 antibody) in RUSH-α5–expressing U2OS cells plated on FN or collagen + biotin (60 min). Grayscale single-channel images and merged images (white, colocalization; blue, nuclei [DAPI]) are shown. **(D)** Representative images of RUSH-α5 and RUSH-CD59 release in U2OS cells co-expressing both constructs and plated on dual-coated micropatterns (alternating FN coating (cyan) and collagen-peptide (GFOGER) (non-fluorescent) lines). Nuclei (blue) are co-labeled. Intensity line profiles generated across the yellow line are displayed relative to the position of the FN-coated micropattern lines. White insets represent regions of interest (ROIs) that are magnified for each channel. FN, fibronectin.

We employed the RUSH (retention using selective hooks) system (Boncompain et al., 2012), which has been previously used to synchronize and study post-Golgi anterograde trafficking of a variety of cargos (Boncompain et al., 2012; Fourriere et al., 2019; Weigel et al., 2021), to control integrin retention and release from the ER (Fig. S1 A). Using this method, we explored, for the first time, the context-dependent traffic of newly synthesized integrins and its implications in cell adhesion and dynamics in real time. Integrin α5β1 is the main FN receptor in many cell types and has been widely studied. Thus far, integrin α5β1 has been tagged on the C-terminal tail, potentially interfering with some established protein–protein interactions (Tuomi et al., 2009; Morse et al., 2014). To identify a suitable alternative tagging site on the receptor’s ectodomain, we examined the published crystal structure of the integrin α5β1 headpiece (Nagae et al., 2012). Given that the N-terminus of the α5 polypeptide is localized between the two integrin subunits, away from the FN ligand binding site, we inserted the IL-2 signal peptide, the streptavidin-binding peptide (SBP) and enhanced green fluorescent protein (EGFP) in this region to generate an SBP-EGFP-integrin α5 construct (henceforth referred to as RUSH-α5) (Fig. 1 A, Fig. S1 A, and Video 1). Computational modeling suggested that while the flexible EGFP C-terminal region (plus linker) is just long enough to allow direct contact between EGFP and the FN ligand binding site (Fig. S1 B and Video 2), EGFP is stably positioned and cannot be displaced within the range of normal physical force. Atomistic simulations were consistent with these observations (Video 3; see Materials and methods for details). Thus, EGFP tagging of the integrin α5 ectodomain does not interfere with FN-binding or α5β1 subunit heterodimerization (Fig. S1 B; and Videos 1, 2, and 3). In cells, RUSH-α5 was retained in the ER when co-expressed with an ER-hook protein composed of the ER-retrieval motif KDEL fused to streptavidin (Fig. 1 B) and released upon biotin addition to be transported to the plasma membrane (Fig. 1 B; Fig. S1, A and C; and Video 4).

**Video 1.** **Rotating model of RUSH-α5 (EGFP-integrin α5)–integrin-β1 heterodimer bound to FN based on (PDB: 7NWL) structure of the heterodimer.** Related to Fig. 1 A.

**Video 2.** **Coarse-grained model simulation depicting pulling of EGFP towards fibronectin binding site and spontaneous relaxation of the final pulled state. (A)** Coarse-grained model, pulling of EGFP towards the FN-binding site. The pulling between the EGFP and the FN-binding site starts from the situation where the C-terminus of the EGFP and the N-terminus of the integrin alpha are <1 nm apart, including the linker. The movie is divided into three parts. It starts with a full rotation around the initial configuration. Then, the pulling is performed (at a constant rate with 1,000 kJ mol^−1^ nm^−2^ at 0.1 nm/ns). Finally, shown is a full rotation around the final state, later used as the first frame for relaxation in Video 2 B. The movie repeats itself in reverse. EGFP is blue, the linker is red, and integrin alpha is orange (one molecule). Integrin beta is yellow, and FN is green. The pulling process spans 8 nm and 80 ns. **(B)** Coarse-grained simulation model, spontaneous (non-biased) relaxation of the final pulled state of Video 2 A. Contacts (<0.6 nm distance) between the EGFP and FN are indicated with magenta (Fig. S1 B). EGFP is blue, the linker is red, and integrin alpha is orange (one molecule). Integrin beta is yellow, and FN is green. The relaxation spans 3,200 ns.

**Video 3.** **Atomistic simulations of EGFP tagged integrin α5β1 bound to fibronectin. (A)** Fully atomistic molecular dynamics simulation of the EGFP attached to the α-subunit of the integrin molecule. The movie depicts a demonstrative simulation of the EGFP (in green) bound to the α-subunit (blue) of integrin. The β-subunit is shown in red color. The FN bound to integrin is shown in gray. No additional forces were applied in this simulation. The simulation is 100 ns long. **(B)** Fully atomistic steered molecular dynamics simulation of the EGFP attached to the α-subunit of the integrin molecule. The movie depicts a demonstrative simulation of the EGFP (in green) bound to the α-subunit (blue) of integrin. The EGFP is pulled towards the FN (gray) binding site with a force of 25 kJ/mol/nm^2^. The β-subunit on integrin is shown in red. The simulation is 100 ns long. **(C)** Fully atomistic steered molecular dynamics simulation of the EGFP attached to the α-subunit of the integrin molecule. The movie depicts a demonstrative simulation of the EGFP (in green) bound to the α-subunit (blue) of integrin. The EGFP is pulled towards the FN (gray) binding site with a force of 50 kJ/mol/nm^2^. The β-subunit on integrin is shown in red. The simulation is 100 ns long.

**Video 4.** **Time lapse spinning-disk confocal imaging of RUSH-α5–expressing U2OS cell plated on FN (10 µg/ml), biotin added after acquisition of time point 0 min.** One frame per minute. Related to Fig. 1 B.

### ECM control of integrin delivery to the plasma membrane

**Figure S2.**
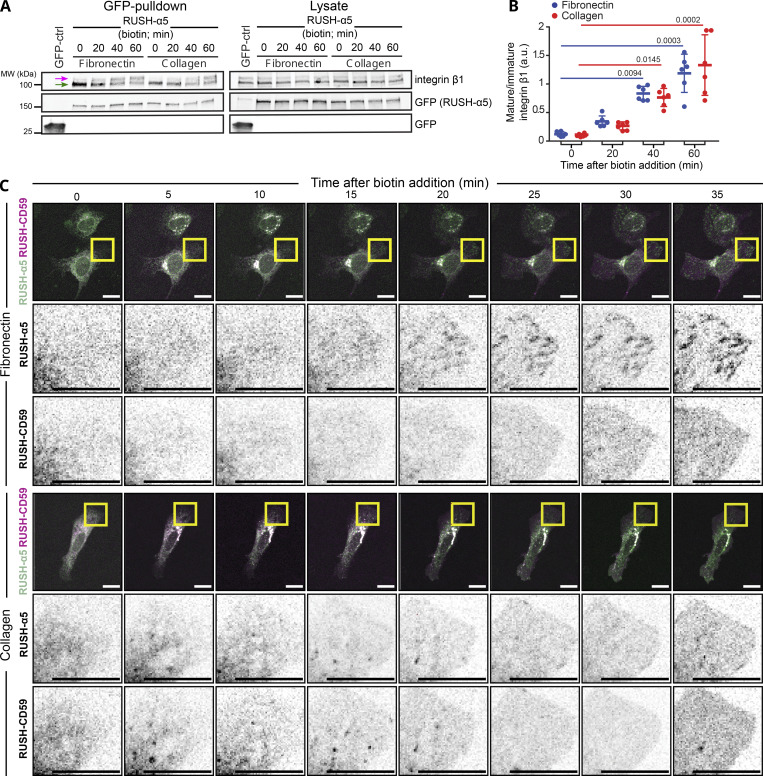
**RUSH-α5 recruitment to adhesions is ligand-dependent. (A)** Representative immunoblot of GFP pulldowns performed in RUSH-α5 or control transfected cells plated on FN or collagen and probed for endogenous integrin β1 and for GFP. Mature (magenta arrow) and immature (green arrow) integrin β1 are indicated. **(B)** Quantification of the relative fraction of mature to immature integrin β1 interacting with RUSH-α5 ± biotin treatment for the indicated times. *N* = 6 independent experiments; data are mean ± SD, One-way ANOVA, Dunn’s multiple comparison test, no significant difference between FN and collagen at all time points. **(C)** Representative images (see Video 5) of U2OS cells co-expressing RUSH-α5 and RUSH-CD59 and plated on FN (top) or collagen (bottom) ± biotin treatment for the indicated times. Insets represent ROIs that are magnified. Scale bars: 20 µm. Source data are available for this figure: SourceData FS2.

In cells, integrin β1 subunits are produced in excess and are transported to the plasma membrane only upon heterodimerization with newly synthesized α-integrins (Heino et al., 1989; De Strooper et al., 1991; Lenter and Vestweber, 1994). To investigate the ability of RUSH-α5 to form functional heterodimers with the β1 subunit, we performed GFP pulldown in cells co-expressing RUSH-α5 and the ER-hook (ER-hook always expressed with RUSH-α5 in all experiments) with and without biotin addition. The EGFP-tagged RUSH-α5 precipitated endogenous β1 integrin. RUSH-α5 interacted with the immature integrin β1 (faster migrating lower band; Fig. S1 D) before release from the ER (0 min biotin), and progressively with the mature integrin β1 after release from the ER following biotin addition (slower migrating upper band; Fig. S1 D). Importantly, RUSH-α5, when released from the ER (60 min biotin), localized to fibrillar and FA-like structures on FN that were positive for active integrin α5 (detected with active integrin α5 conformation-specific SNAKA51 antibody) (Fig. 1 C). In contrast, RUSH-α5 displayed a clearly reduced localization to adhesion structures along with a more diffuse localization pattern in cells plated on collagen and treated with biotin (Fig. 1 C). The ECM ligand did not dramatically influence integrin heterodimer maturation (increasing ratio of mature to immature integrin β1) as it was not significantly faster on FN than on collagen 20 and 40 min after biotin addition (Fig. S2, A and B).

Our real-time measurements revealed, however, an interesting and unexpected feature of integrin maturation and delivery. Metabolic labeling studies have indicated receptor maturation kinetics exceeding 1 h for the total integrin β1 cellular pool (Heino et al., 1989; Tiwari et al., 2011). These pulse-labeling methods analyze synthesis, folding and secretion of integrins, and cannot be directly compared with the RUSH-method. However, our data here reveals that α5β1-integrin secretion and maturation can be detected already 20 min after integrin release from the ER (Fig. S2, A and B). Taken together, these data indicate that RUSH-α5 forms a functional heterodimer with endogenous integrin β1, undergoes ligand-specific activation on FN, and the dynamics and kinetics of the newly synthesized integrin can be analyzed using this method.

**Figure 2.**
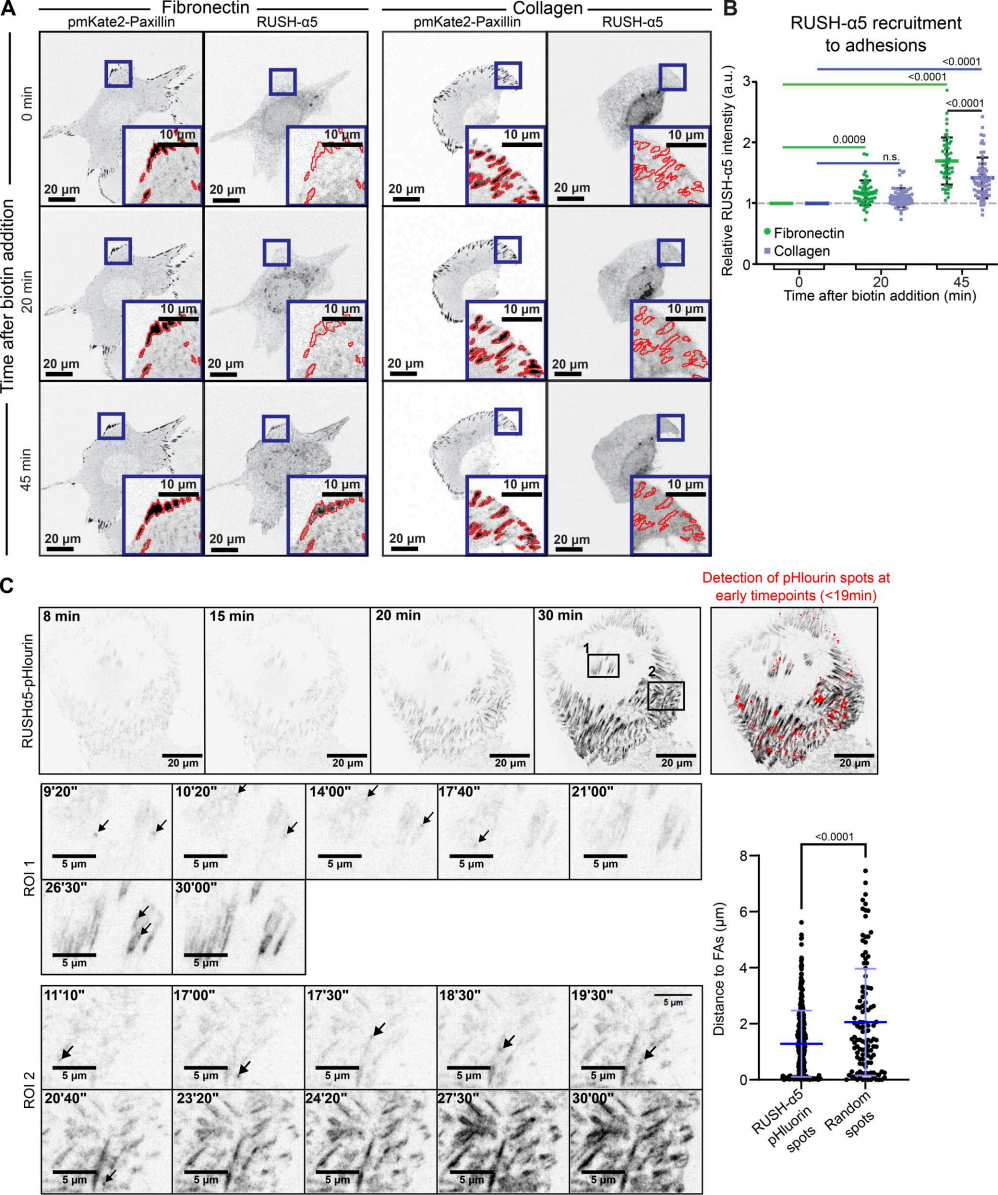
**RUSH-α5 delivery to FAs. (A)** Representative immunofluorescence images of U2OS cells co-expressing RUSH-α5 and pmKate2-Paxillin plated on FN or collagen ± biotin treatment for the indicated times. Insets represent ROIs that are magnified and show paxillin-segmented adhesions (red outlines). **(B)** Quantification of the relative mean intensity of RUSH-α5 in segmented adhesions/cell ± biotin treatment for the indicated times. Data are mean ± SD; *n* = 64 cells on collagen, 50 cells on FN, pooled from three independent experiments; One-way ANOVA, Holm-Šídák’s multiple comparison test; data distribution was assumed to be normal but this was not formally tested. **(C)** TIRF imaging of U2OS cells expressing RUSH-α5-pHluorin on a FN-coated surface after biotin release at T = 0. The arrows indicate exocytosis events. Exocytosis events were detected by performing a ratiometric analysis, which consisted of dividing each frame by the previous. All detected events before 19 min of release are indicated in red. The graph indicates the distance between the exocytosis events and the nearest FA segmented on the last frame of Video 6 (37 min after release), compared to the distance of random dots to FAs, showing that the localization to FAs is not random. Individual measurements and the mean ± SD are represented. Unpaired *T* test. RUSH-α5-pHluorin spots *n* = 336 spots, random spots *n* = 116 spots from one experiment.

**Figure S3.**
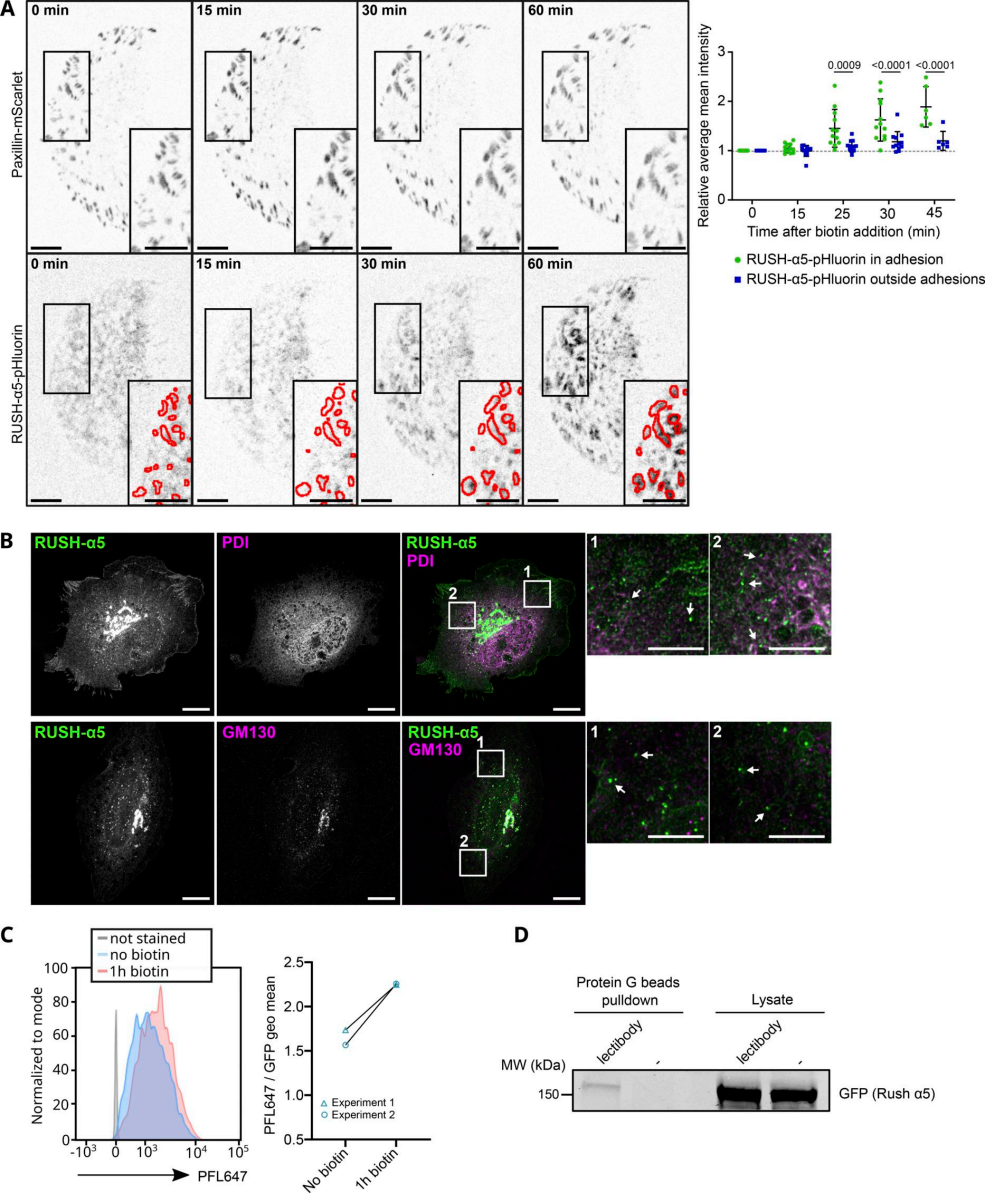
**RUSH-α5 delivery and localization following release. (A)** RUSH-α5-pHluorin released in U2OS co-expressing Paxillin-mScarlet on FN- and anti-GFP antibody-coated surfaces. The intensity of RUSH-α5-pHluorin signal was quantified in and outside adhesions (paxillin positive, represented in the insets). Data are mean ± SD, *N* = 12 cells (*N* = 6 cells from 1 experiment for T = 45 min), pooled from 2 independent experiments. Ordinary one-way Anova with Holm-Šídák’s multiple comparisons test; data distribution was assumed to be normal but this was not formally tested. Scale bars: 10 µm (main and insets). **(B)** High resolution imaging of RUSH-α5 after 15 min of release in U2OS. PDI (ER marker) or GM130 (Golgi marker) are co-stained. Arrows in the insets indicate RUSH-α5 positive vesicles. Scale bar: 10 µm (main), 5 µm (insets). **(C and D)** High-mannose integrin-α5 is delivered to the cell surface. **(C)** Flow cytometry analysis of high-mannose proteins at the cell surface detected with the fluorescent lectin PFL647 in U2OS cells expressing RUSH-α5, without release and 1 h after release. The left panel shows histograms of one experiment, the right panel shows the geometric fluorescence mean of the PFL647 signal for individual experiments (*N* = 2 independent experiments). **(D)** U2OS expressing RUSH-α5 were labeled at their surface after 1 h release with a lectibody specifically recognizing high-mannose proteins. The lectibody was then pulled down by protein G beads. This Western blot shows GFP detection in the pull-down, indicating the presence of high-mannose RUSH-α5 at the cell surface after release. Representative of *N* = 3 independent experiments. Source data are available for this figure: SourceData FS3.

To explore the kinetics of the biosynthetic delivery of integrins in more detail, we performed time-lapse imaging of RUSH-α5 release in cells plated on collagen and FN, comparing it with the dynamics of a co-expressed SBP- and mCherry-tagged control cargo protein CD59 (glycosylphosphatidylinositol-anchored proteins cluster of differentiation 59) (Boncompain et al., 2012), henceforth called RUSH-CD59. On both ECMs, RUSH-α5 and RUSH-CD59 were localized to the ER in the absence of biotin, and following biotin addition were released and transported to the Golgi, residing there for ∼15 min (Fig. S2 C), in line with previous reports for CD59 (Boncompain et al., 2012; Fourriere et al., 2019). After 20 min, RUSH-α5 was predominantly localized on adhesion-like structures on FN whereas on collagen it was diffusely distributed on the plasma membrane (Fig. S2 C and Video 5). In contrast, the RUSH-CD59 construct behaved similarly on both FN and collagen (Fig. S2 C and Video 5), indicating that the observed differences in RUSH-α5 localization in cells on FN and collagen were ligand-receptor specific. This was further validated by plating RUSH-α5 and RUSH-CD59 co-transfected cells on dual-coated micropatterns (Isomursu et al., 2024) with alternating lines of FN and GFOGER (a synthetic collagen peptide with high affinity for collagen-binding integrins [Zhang et al., 2003]). Following release (20-min biotin), RUSH-α5 localized predominantly to the cell edges, showing enriched clustering on FN-coated lines, compared to GFOGER-coated lines (Fig. 1 D). In contrast, RUSH-CD59 localization was independent of ligand coating. This implies that the integrin is largely delivered directly to the FN areas. However, as we are focusing only on the ventral surface of cells, we cannot exclude the possibility that integrin α5 could be delivered first on the dorsal surface of the cells and then diffuse to the ventral surface into FAs. Next, we examined RUSH-α5 targeting to adhesions by co-expressing pmKate2-paxillin as a FA marker. RUSH-α5 was localized to pmKate2-paxillin-positive adhesions as early as 20 min following release with biotin, and this localization increased over time (Fig. 2, A and B). On collagen, RUSH-α5 localization to adhesions was significantly lower compared to FN (Fig. 2, A and B), with the increase in intensity at the later time point most likely reflecting the increased presence of the diffused receptor on the membrane. We then replaced the EGFP in RUSH-α5 with pHluorin, a pH sensitive GFP (Miesenböck et al., 1998), to image RUSH-α5 cell surface delivery. The pHluorin GFP is non-fluorescent in acidic secretory vesicles and becomes fluorescent upon cell surface exposure. To detect the localization of RUSH-α5 secretion, we prevented receptor diffusion after ventral plasma membrane delivery by employing the selective protein immobilization (SPI) method (Fourriere et al., 2019). The ECM proteins were coated alongside anti-GFP antibodies, which bind to the GFP moiety in RUSH-α5 and trap the receptor when it is delivered to the cell surface. In cells co-transfected with RUSH-α5-pHluorin and paxillin-mScarlet, we observed a specific increase in pHluorin signal in FAs over time (Fig. S3 A). Moreover, TIRF live imaging of the RUSH-α5-pHluorin showed fluorescent flashes corresponding to RUSH-α5 surface delivery. These exocytosis events were significantly closer to FAs compared to those of randomly generated points (Fig. 2 C and Video 6). These results indicate that RUSH-α5 is predominantly delivered to FA sites on FN. In addition, it is possible that some of the integrin is transported elsewhere on the plasma membrane and rapidly diffuses to the FN-specific adhesions further contributing to FA formation by the newly synthesized integrin.

**Video 5.** **Time lapse spinning-disk confocal imaging of U2OS cells co-expressing RUSH-α5 (green) and RUSH-CD59 (magenta) and plated on FN (left, 10 µg/ml) or collagen (right, 10 µg/ml), biotin added after acquisition of time point 0 min.** One frame per 30 s. Related to Fig. S2 C.

**Video 6.** **Time lapse TIRF imaging of U2OS expressing RUSH-α5-pHluorin plated on FN-coated surface.** Left: RUSH-α5-pHluorin. Right: ratiometric analysis, the exocytosis spots appear in yellow. Scale bar: 20 µm. One frame per 10 s. Related to Fig. 2 C.

### Polarized delivery of new integrin to the cell protruding edge

**Figure 3.**
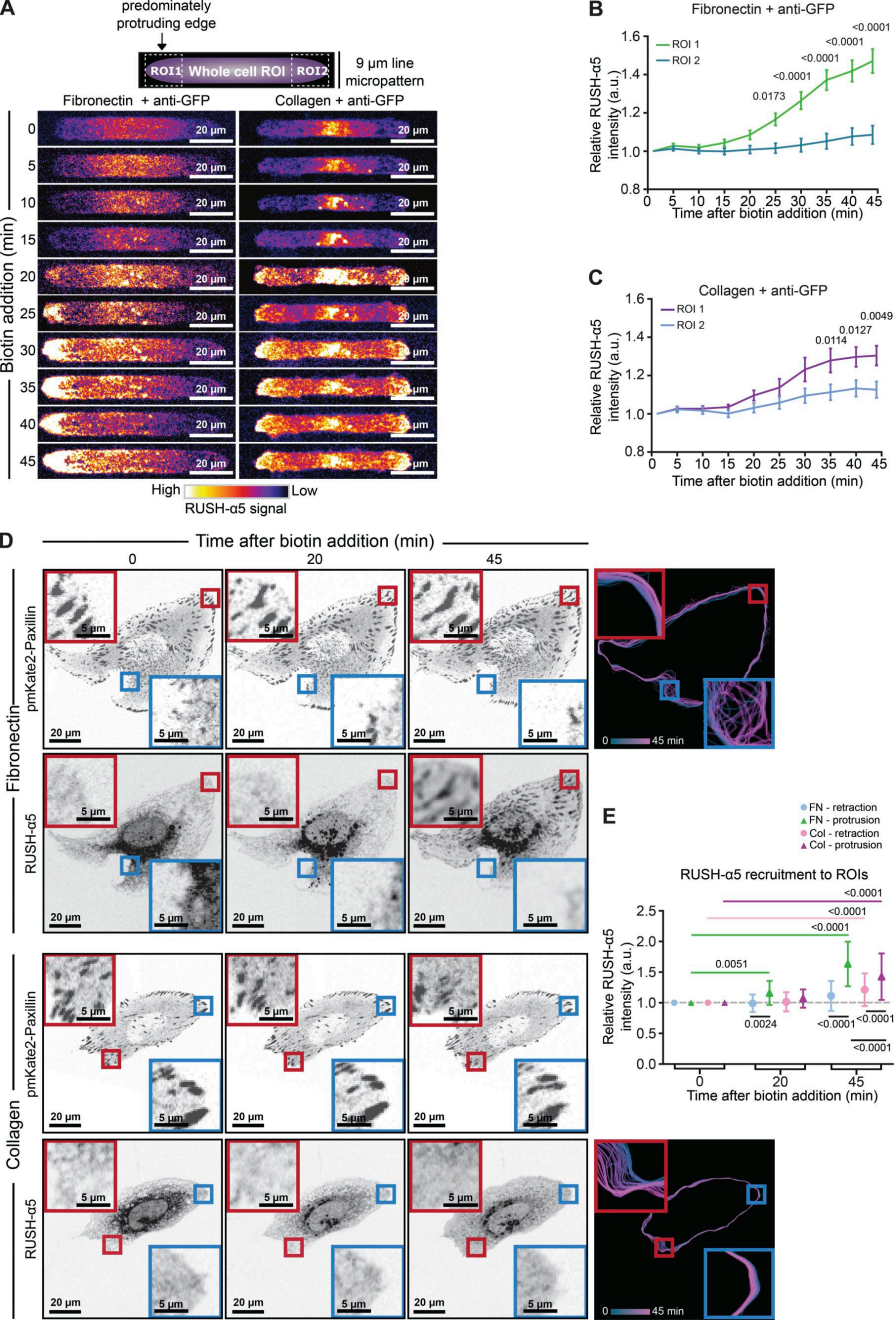
**Polarized delivery of newly synthesized integrin to the cell protruding edge. (A–C)** RUSH-α5 intensity in U2OS cells plated on 9 µm-wide micropatterns coated with FN and anti-GFP or collagen and anti-GFP ± biotin treatment for the indicated times was analyzed at both cell edges (the predominantly protruding edge was denoted ROI1 and the other edge ROI2; Videos 7 and 8). Representative intensity coded images (A) and quantification of RUSH-α5 release on FN (B; Video 7) and collagen (C; Video 8) (normalized first to the total intensity of the cell and then to 0 min biotin) are shown. Data are mean ± SEM. **(D)** Representative images and spatiotemporal track maps of cell edge contours over time in U2OS cells expressing RUSH-α5 ± biotin treatment for the indicated times. Red insets represent protruding ROIs that are magnified. Blue insets represent retracting ROIs that are magnified. Spatiotemporal track maps: blue colors represent early time points and magenta colors represent late time points in the time-lapse series. **(E)** Quantifications of RUSH-α5 intensity in ROIs (retracting or protruding areas determined from spatiotemporal track maps). Data are mean ± SD. **(B and C)***N* = 33 cells on FN and 38 cells on collagen, pooled from three independent experiments, two-way ANOVA, Holm-Šídák’s multiple comparison test. **(E)***N* = 53 cells on collagen, 49 cells on FN, pooled from three independent experiments; one-way ANOVA, Holm-Šídák’s multiple comparisons test; data distribution was assumed to be normal but this was not formally tested.

We then investigated if the localization of newly synthesized integrins is polarized. First, we plated RUSH-α5 transfected cells on 9 µm-wide collagen or FN micropatterned lines (in combination with the GFP-SPI method to prevent RUSH-α5 diffusion after delivery), shown previously to support front-rear cell polarity of integrins (Shafaq-Zadah et al., 2016). Time-lapse imaging revealed a significant increase in RUSH-α5 intensity on FN over time preferentially at the protruding edge of the cell (region of interest 1; ROI1) (Fig. 3, A and B; and Video 7) whereas, on collagen lines, the difference in RUSH-α5 localization between the 2 cell edges was modest and apparent only at later time points (Fig. 3, A and C; and Video 8). In line with these data, RUSH-α5 delivery was also polarized on FN in unconstrained cells, occurring significantly more in protruding regions, whereas on collagen RUSH-α5 intensity increased both in retracting and protruding regions (protruding or retracting areas of the cells defined based on spatiotemporal track maps (Baniukiewicz et al., 2018), generated from paxillin images) (Fig. 3, D and E). These data indicate that the plasma membrane delivery of newly synthesized integrins is sensitive to ECM ligand engagement and the cell front-rear polarity.

**Video 7.** **Time lapse spinning-disk confocal imaging of U2OS expressing RUSH-α5 plated on 9 µm-wide FN and anti-GFP-coated micropattern lines.** Biotin added after acquisition of time point 0 min. One frame per minute. Related to Fig. 3, A and B.

**Video 8.** **Time lapse imaging of spinning-disk confocal U2OS expressing RUSH-α5 plated on 9 µm-wide collagen and anti-GFP-coated micropattern lines.** Biotin added after acquisition of time point 0 min. One frame per minute. Related to Fig. 3, A and C.

### Rapid, adhesion-dependent delivery of RUSH-α5

**Figure 4.**
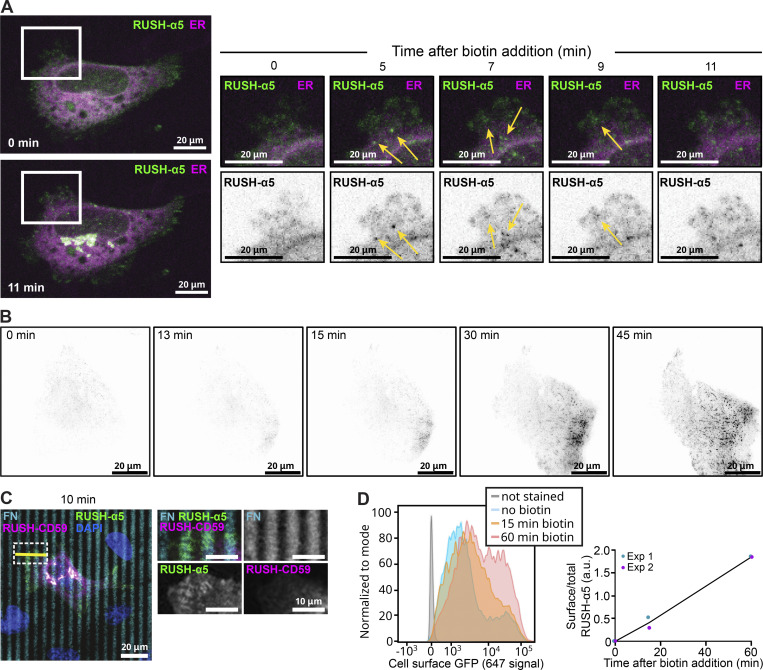
**Early delivery of RUSH-α5 to the cell surface. (A)** Representative immunofluorescence images of U2OS cells co-expressing RUSH-α5 (green) and the ER marker ERoxBFP (magenta) plated on FN (10 µg/ml) ± biotin treatment for the indicated times. Arrows indicate rapidly budding RUSH-α5–positive vesicles adjacent to cell protrusions (≤15 min after release) (see also Video 9). **(B)** TIRF imaging of RUSH-α5 after release (0 min). The polarized delivery to the cell surface at the protruding area can be observed from 15 min after release (see also Video 10). **(C)** Representative images of RUSH-α5 (green) and RUSH-CD59 (magenta) release in U2OS cells co-expressing both constructs and plated on dual-coated micropatterns (alternating FN coating (cyan) and collagen-peptide (GFOGER) (non-fluorescent) lines). Nuclei (blue) are co-labeled. White insets represent ROIs that are magnified for each channel. FN, fibronectin. **(D)** Flow cytometry analysis of cell surface RUSH-α5 levels (detected with the anti-GFP-AF647 antibody) in RUSH-α5–expressing U2OS cells ± biotin. Representative histograms and quantification from two independent experiments of cell surface GFP (ratio of the geometric means of the surface signal divided by the total GFP signal, normalized by subtracting the 0 min value) are shown.

While RUSH-α5 was predominantly trafficked to the plasma membrane via the Golgi complex conventional secretion pathway, a process that takes more than 20 min, surprisingly, some RUSH-α5–positive vesicles were evident earlier (around 10 min) within the vicinity of the plasma membrane (Fig. 4 A and Video 9). This was also apparent using TIRF live imaging where the RUSH-α5 signal was detected at the cell-ECM interface already at 13 min post release. After 15 min surface delivery was clearly polarized to the cell leading-edge protruding area (Fig. 4 B and Video 10). Moreover, using pHluorin-RUSH-α5, we were able to observe exocytosis events from 10 min after release (Fig. 2 C and Video 6). This unexpected early plasma membrane delivered integrin localized to FN-line micropatterns unlike the RUSH-CD59 construct (Fig. 4 C). Cell surface delivery of RUSH-α5 was also detected with flow cytometry 15 min after biotin addition with a steady increase up to 1 h (Fig. 4 D). Live imaging of RUSH-α5 together with an ER marker (ERoxBFP) revealed RUSH-α5 puncta outside of the ER and being trafficked in close proximity to FAs at very early time points (Video 9). High resolution confocal imaging of cells transfected with RUSH-α5 after 10 min of release showed no obvious overlap between RUSH-α5 vesicles and Golgi or ER makers (Fig. S3 B). These observations suggest that at least some of the RUSH-α5 is delivered to the plasma membrane without going through the Golgi apparatus.

**Video 9.** **Time lapse spinning-disk confocal imaging of U2OS cells co-expressing RUSH-α5 (green) and the ER marker ERoxBFP (magenta) plated on FN (10 µg/ml), biotin added after acquisition of time point 0 min.** One frame per 30 s. Related to Fig. 4 A.

**Video 10.** **Time lapse imaging of U2OS cells expressing RUSH-α5 plated on FN (10 µg/ml), biotin added after acquisition of time point 0 min, imaged by TIRF microscopy.** One frame per 30 s. Related to Fig. 4 B.

### Integrin rapid delivery bypasses conventional Golgi secretion

**Figure 5.**
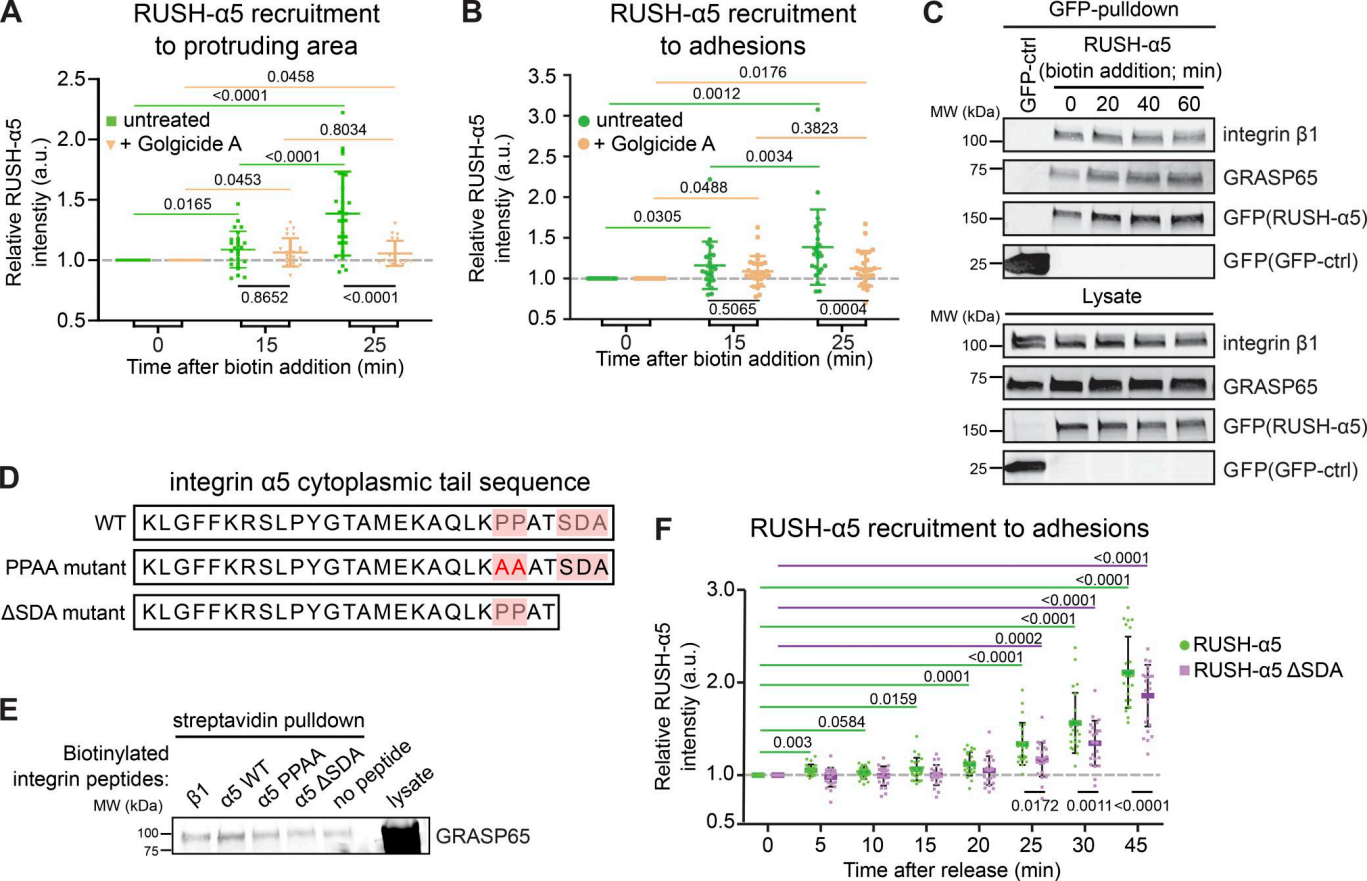
**Golgi bypass early delivery of RUSH-α5 requires the integrin-α5 PDZ-binding motif. (A and B)** Quantification of relative RUSH-α5 recruitment to protruding areas (A) or adhesions (B) in U2OS cells expressing RUSH-α5 ± biotin treatment for the indicated times with or without Golgicide A (10 µM). **(C)** Representative immunoblot of GFP pulldowns from RUSH-α5 or GFP control transfected cells plated on FN and probed for GFP and endogenous GRASP65. *N* = 3 independent experiments. **(D)** Amino acid sequence of the integrin α5 tail highlighting the canonical PDZ-binding motif (SDA) and the two proline residues critical for the formation of the non-canonical PDZ-binding motif. The mutations of these sites used in our experiments are indicated below. **(E)** Representative streptavidin pulldowns of the indicated biotinylated recombinant integrin peptides incubated with cell lysates collected from CHO cells overexpressing GFP-GRASP65. A representative immunoblot probed for GRASP65 (note, two bands are present in the lysate: upper, GFP-GRASP65; lower, endogenous GRASP65; GFP-GRASP65 is apparent in the pulldown). *N* = 3 independent experiments. **(F)** Quantification of RUSH-α5 or RUSH-α5 ΔSDA recruitment to adhesions in U2OS cells ± biotin treatment for the indicated times. All data are mean ± SD. **(A and B)** One-way ANOVA with Tukey’s multiple comparison test for comparing time points, one-way ANOVA with Holm-Šídák’s multiple comparisons test for comparing untreated and Golgicide A, data distribution was assumed to be normal but this was not formally tested. **(A)***N* = 26 cells RUSH-α5, *N* = 22 cells RUSH-α5 Golgicide A, pooled from three independent experiments. **(B)***N* = 24 cells RUSH-α5, *N* = 27 cells RUSH-α5 Golgicide A, pooled from three independent experiments. **(F)** One sample t test to compare time points with T = 0, ordinary one-way ANOVA with Holm-Šídák’s multiple comparisons test to compare RUSH-α5 and RUSH-α5 ΔSDA, data distribution was assumed to be normal but this was not formally tested. *N* = 23 cells RUSH-α5, *N* = 23 cells RUSH-α5 ΔSDA, pooled from two independent experiments. Source data are available for this figure: SourceData F5.

These data prompted us to hypothesize that a small proportion of integrins could undergo unconventional protein secretion (UPS), a process where secretory proteins are transported from the ER to the plasma membrane without entering the Golgi complex (Rabouille, 2017). Conventional Golgi secretion is inhibited by Golgicide A (Sáenz et al., 2009). Therefore, we treated cells with Golgicide A to investigate the relative contribution of the Golgi complex to RUSH-α5 trafficking. As expected, the delivery of the majority of RUSH-α5, 25 min post-biotin addition, was significantly inhibited by Golgicide A (Fig. 5, A and B). However, both control and Golgicide A-treated cells showed a small initial increase in RUSH-α5 recruitment to protruding areas of the cell and to adhesions 15 min after release (Fig. 5, A and B), suggesting that a small fraction of newly synthesized integrins may be secreted to FAs via a mechanism that bypasses conventional Golgi secretion.

In normal cells, high-mannose-type glycans are predominantly localized to the ER, and undergo enzymatic processing and maturation into complex glycoforms in the Golgi during conventional secretion (Ungar, 2009). However, several cancer types and cancer patient samples show significantly increased proportions of high-mannose glycans on the cell surface (Liu et al., 2013; Zhang et al., 2015) and unbiased probing with high-mannose binding lectins have shown that integrins are abundant in this receptor pool (Oh et al., 2022). This prompted us to investigate the presence of high-mannose glycans at the cell surface. We used the fluorescently labeled *Pseudomonas fluorescens* Pf0-1lectin (PFL), which specifically binds to high-mannose glycans (Sato et al., 2012) to stain the surface of RUSH-α5 transfected cells before and after release (1 h). We observed increased PFL staining after release, indicating delivery of high-mannose proteins to the cell surface (Fig. S3 C). To explore this further, we took an orthogonal approach and labeled the surface of RUSH-α5 cells after biotin addition (1 h release) with a high-mannose specific antibody-like lectibody molecule (Oh et al., 2022; Hamorsky et al., 2019) and subsequently performed lectibody pulldowns. We detected RUSH-α5 in these pulldowns, indicative of high-mannose α5-integrin expression on the cell surface (Fig. S3 D).

**Figure S4.**
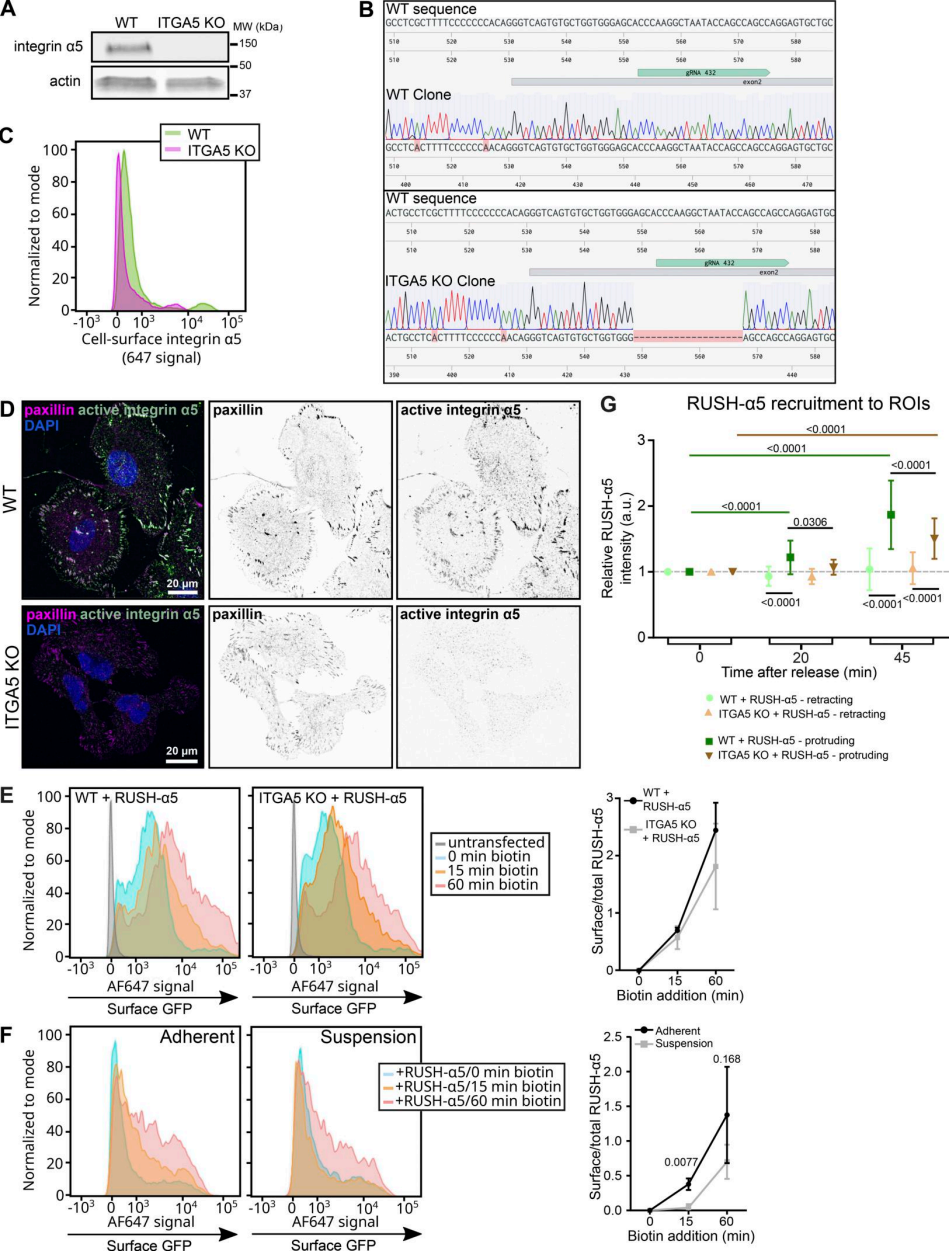
**Early release of RUSH-α5 is adhesion dependent and polarized recruitment to protrusions is supported by endogenous integrin α5. (A–D)** Validation of ITGA5 CRISPR-Cas9 KO U2OS cells. **(A)** Western blot analysis of WT and ITGA5 KO single cell clones showing the efficiency of the CRISPR-Cas9 ITGA5 KO in U2OS cells. **(B)** Genome sequence alignment of U2OS WT and ITGA5 KO clones with the ITGA5 WT sequence. The targeted exon and the gRNA used for CRISPR KO positions are indicated. **(C)** Representative flow cytometry analysis of cell surface integrin α5 in U2OS WT and ITGA5 KO clones. **(D)** Images of WT and ITGA5 KO U2OS clones stained for active integrin α5 (SNAKA51) and paxillin. Scale bar: 20 µm. **(E)** Flow cytometry analysis of cell surface RUSH-α5 levels (detected with the anti-GFP-AF647 antibody) in WT and ITGA5 KO U2OS cells transfected with RUSH-α5 ± biotin treatment for the indicated times. Representative histograms and quantification of cell surface GFP (ratio of the geometric means of the surface signal divided by the total GFP signal, normalized by subtracting the 0 min value) are shown. Data are mean ± SD; *N* = 3 independent experiments. The two-tailed paired *t* test showed no significant differences between WT and ITGA5 KO. Data distribution was assumed to be normal but this was not formally tested. **(F)** Flow cytometry analysis of cell surface RUSH-α5 levels in adherent versus suspension U2OS cells expressing RUSH-α5 ± biotin treatment for the indicated times. Representative histograms and quantification of cell surface GFP analyzed as in E are shown. Data are mean ± SD; *N* = 3 independent experiments. The two-tailed paired t test, data distribution was assumed to be normal but this was not formally tested. **(G)** Quantifications of RUSH-α5 intensity in ROIs (retracting or protruding areas) in WT and ITGA5 KO U2OS cells ± biotin treatment for the indicated times. One-way ANOVA, Holm-Šídák’s multiple comparison test, data distribution was assumed to be normal but this was not formally tested. Data are mean ± SD; *N* = 59 WT cells, 53 ITGA5 KO cells, pooled from three independent experiments. Source data are available for this figure: SourceData FS4.

While cell surface high-mannose bearing integrins have been reported in cancer cells (Liu et al., 2013; Zhang et al., 2015), insights into the relevance of Golgi bypass has been limited to the integrin αPS1 subunit during Drosophila follicle epithelium development, stimulated by mechanical stress (Schotman et al., 2009). This prompted us to investigate whether early secretion of newly synthetized integrins in mammalian cells is linked to cell adhesion and adhesion-induced mechanics. First, we explored whether preexisting endogenous integrin α5 adhesions are involved. We knocked-out endogenous integrin α5 (ITGA5) (Fig. S4, A–D) and compared RUSH-α5 release in wild-type (WT) and knockout (KO) cells after 15 and 60 min biotin addition (note that U2OS cells have other FN-binding integrins in addition to integrin α5 and thus the KO cells adhere to FN similarly to control cells). RUSH-α5 delivery to the cell surface was comparable between WT and ITGA5 KO cells (Fig. S4 E). Thus, the early secretion of newly synthesized integrin α5 is not dependent on the localization of the endogenous protein already at the cell surface. However, the early delivery of RUSH-α5 to the plasma membrane was dependent on cell-ECM adhesion, as we did not detect RUSH-α5 at the cell surface after 15 min biotin addition in suspension cells (Fig. S4 F). Adhesion was not required for slower integrin secretion (60 min biotin) via the conventional pathway (Fig. S4 F). These data are consistent with cell adhesion and perhaps active spreading/protrusions acting as necessary triggers for early secretion of integrin α5 to the cell surface. Even though the early delivery of RUSH-α5 was not, as such, dependent on endogenous integrin α5, polarized RUSH-α5 localization to cell protruding areas was significantly higher in WT compared to ITGA5 KO cells (Fig. S4 G), suggesting that polarized localization of the newly synthesized integrin is orchestrated by existing adhesions and contributes to rapid alterations in cell shape.

### Unconventional RUSH-α5 secretion is dependent on the integrin-tail PDZ-binding motif

**Figure S5.**
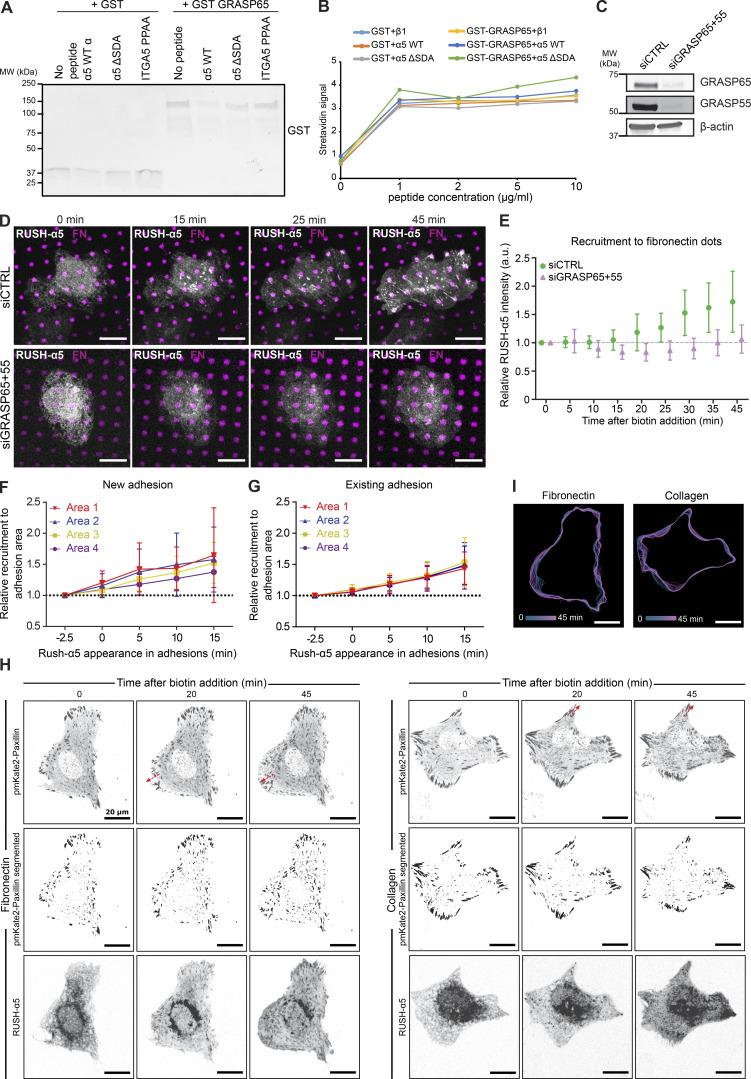
**Early release of RUSH-α5 is sensitive to GRASP silencing. (A)** Immunoblot of GST pull-downs of recombinant integrin α5 WT or mutant (ΔSDA or PPAA) peptides incubated with GST alone or recombinant GST-GRASP65. No enrichment of GST-GRASP65 signal over background (no peptide beads) is detected with integrin α5 WT peptide, indicating that the integrin α5 peptides do not interact with recombinant purified GST-GRASP65. **(B)** ELISA assay detecting biotinylated recombinant integrin α5 WT or ΔSDA or PPAA mutant with HRP-streptavidin incubated on wells coated with GST alone or GST-GRASP65. No direct interaction between GRASP65 and integrin α5 WT peptide was detected. **(C)** Immunoblot of lysates collected from control-silenced or GRASP65 and GRASP55-silenced U2OS cells used in D, E, probed for GRASP65 and GRASP55. β-actin was probed as a loading control. **(D)** Representative immunofluorescence images of control-silenced or GRASP65 and GRASP55-silenced U2OS cells expressing RUSH-α5 and plated on dual-coated micropatterns (magenta dots, FN; non-fluorescent regions, collagen peptide GFOGER). **(E)** Relative recruitment of RUSH-α5 in control- or GRASP65- and GRASP55-silenced U2OS cells to FN dots within the cell boundary. Data are mean ± SD; *n* = 9 siCTRL cells, 11 siGRASP cells (36 and 44 dots, respectively) from one experiment. **(F and G)** Quantification of RUSH-α5 intensity in the four areas relative to signal intensity in the respective area 2.5 min prior to RUSH-α5 appearance in (F) new adhesions or (G) already existing adhesions (determined from the time-lapse images) on FN- and anti-GFP antibody-coated surfaces. Adhesions close to the cell edge and with a minimum lifetime of 15 min were analyzed and changes of RUSH-α5 intensity were plotted over time in the indicated areas ranging from distal to proximal to the cell body. Data are mean ± SD; One independent experiment 9 adhesions from 6 cells on 2 coverslips (F) and one independent experiment 9 adhesions from 5 cells on 2 coverslips (G). **(H and I)** Representative images (H) and (I) track maps related to Fig. 6 I. Red arrows indicate direction of adhesion growth. Scale bars: 20 µm. Source data are available for this figure: SourceData FS5.

The two mammalian Golgi reassembly-stacking protein (GRASP) homologs, GRASP55 and GRASP65, mediate UPS of transmembrane proteins via PDZ domain-mediated interactions with cargo proteins (Gee et al., 2018; Kim et al., 2016). The integrin α5 cytoplasmic domain harbors two distinct PDZ-binding motifs: a classical C-terminal Ser-Asp-Ala (SDA) sequence (El Mourabit et al., 2002) and a non-canonical motif generated by two prolines (PP), which induce an internal β-hairpin that functions as a PDZ-recognition motif (Tuomi et al., 2009). To test whether integrin α5 associates with GRASPs, we performed GFP pulldowns in cells expressing RUSH-α5. In addition to integrin β1, we detected endogenous GRASP65 co-precipitating with RUSH-α5 (Fig. 5 C). Further pulldown experiments with biotinylated peptides corresponding to the C-terminal part of the integrin α5 WT tail or integrin α5 tails with mutations in the non-canonical PDZ-binding motif (PPAA peptide) or deletion of the canonical PDZ-binding motif (ΔSDA) (Fig. 5, D and E) indicated that GRASP65-integrin α5 association may require the SDA sequence (Fig. 5 E), in accordance with the ability of GRASP65 to facilitate UPS of ER-resident cargo containing PDZ-binding motifs and regulate N-linked glycosylation in the ER (Xiang et al., 2013). However, no direct interaction between recombinant GRASP65 and integrin α5 was observed by GST pulldown (Fig. S5 A) or ELISA (Fig. S5 B). This indicates that the association between integrin α5 and GRASP65 is indirect, requires additional co-factors or is sensitive to post-translational modifications.

We then explored the involvement of GRASP and the integrin tail in the early delivery of RUSH-α5. siRNA-mediated silencing of GRASP65 and GRASP55 (Fig. S5 C) inhibited RUSH-α5 delivery to FN spots on dual-coated (FN and GFOGER) micropatterns at 10–15 min post biotin addition (Fig. S5, D and E). However, the recruitment of RUSH-α5 to FN dots remained low 25 min after release, possibly due to GRASP silencing interfering with Golgi function (Xiang and Wang, 2010). Furthermore, GRASP depletion has been shown to downregulate integrin α5β1 protein levels and could also affect the lifetime of our exogenous construct (Ahat et al., 2019). To overcome these complications, we generated a RUSH-α5 construct lacking the potential integrin α5 GRASP65-binding SDA sequence (RUSH-α5-ΔSDA) and performed time-lapse imaging. RUSH-α5-ΔSDA recruitment to adhesions was delayed compared to cells expressing full-length RUSH-α5 (25 versus 5 min) (Fig. 5 F). Even after 45 min, RUSH-α5-ΔSDA accumulation in adhesions was diminished, consistent with unconventional secretion accounting for a small part of the overall integrin biosynthetic delivery in cells.

### New integrins are delivered to FAs to drive adhesion growth

**Figure 6.**
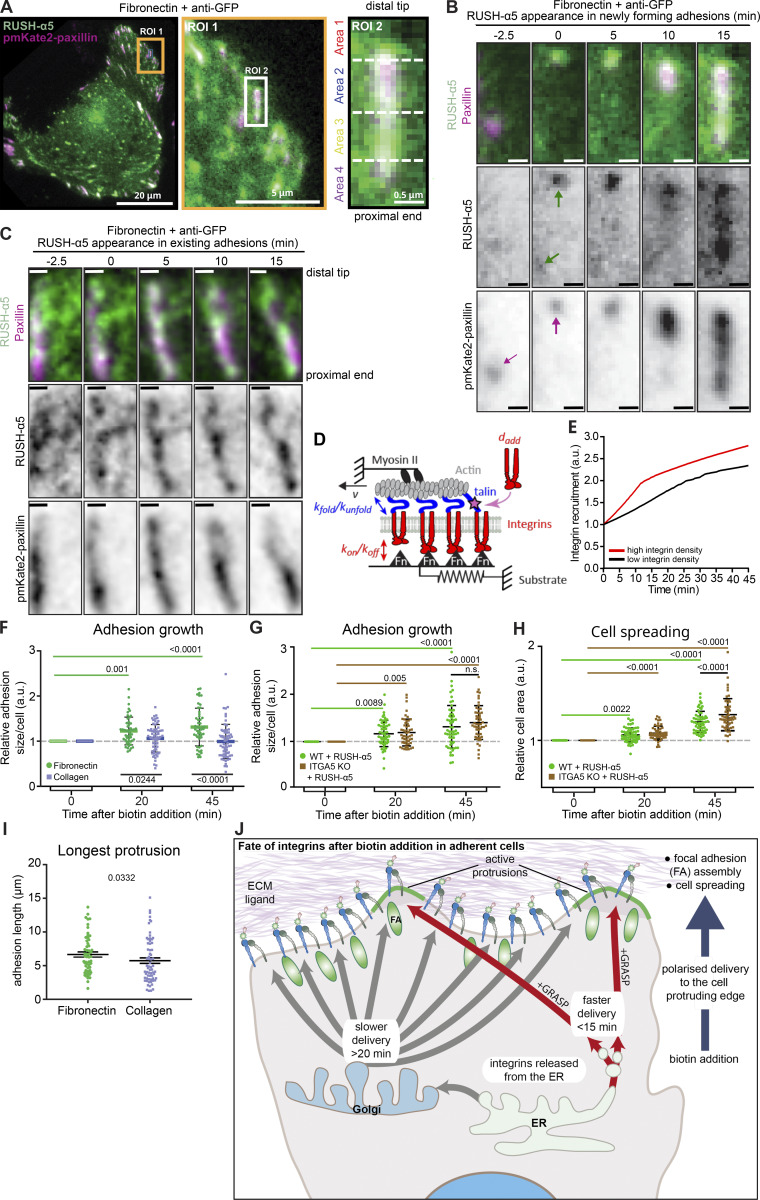
**RUSH-α5 is delivered to the tip of adhesions and mediates adhesion growth. (A and B)** Representative immunofluorescence image of U2OS cells expressing RUSH-α5 and pmKate2-Paxillin (white, colocalization) plated on FN (10 µg/ml) and anti-GFP (2.5 µg/ml; to trap cell surface RUSH-α5 at the point of delivery). Insets represent ROIs that are magnified. ROI2 is a FA demarcated into four equal areas for analysis and is further magnified in B. Scale bars 20 µm (whole cell image), 5 µm (ROI1), and 0.5 µm (ROI2 and B). **(C)** Representative image of an already established FA where RUSH-α5 delivery was quantified. Scale bar 0.5 µm. **(D)** Cartoon showing clutch model elements. Myosin motors pull on actin filaments with a speed *v*. This applies force to a substrate via integrins and adapter proteins (talin). The effect of force regulates the unbinding rates from integrins to the substrate (*k*_*off*_) and the folding/unfolding rates of talin (*k*_*fold*_/*k*_*unfold*_). When talin unfolds, adhesion reinforcement is assumed to happen, which is modeled by an increase in integrin density with value *d*_*add*_. Changes in integrin availability are modeled by changing the parameter *d*_*add*_. **(E)** Model prediction of adhesion growth with time for conditions in which integrin availability is low (*d*_*add*_ = 0.005 integrins/μm^2^) or high (*d*_*add*_ = 0.01 integrins/μm^2^). Adhesion growth (y-axis) is modeled through integrin density, which is plotted normalized to the starting value. **(F)** Quantification of adhesion growth in U2OS cells expressing RUSH-α5 and plated on FN or collagen ± biotin treatment for the indicated times. Shown are the relative sums of segmented adhesion area/cell. Data are mean ± SD. **(G)** Quantification of adhesion growth in WT and ITGA5 KO U2OS cells expressing RUSH-α5 ± biotin treatment for the indicated times. Shown are the relative sums of segmented adhesion area/cell. Data are mean ± SD. **(H)** Quantification of cell spreading in WT and ITGA5 KO cells expressing RUSH-α5 ± biotin treatment for the indicated times. Data are mean ± SEM. **(I)** Quantification of the length of the longest protrusion (extending furthest from the initial plasma membrane localization during imaging) formed per cell after 45 min of biotin. Data are mean ± SEM. **(J)** Schematic depiction of the regulation of cell dynamics by transport of integrins through the biosynthetic pathway. Adhesion and cell spreading-dependent delivery of integrin from the ER is detected rapidly after release in cell protrusions. Canonical Golgi-dependent delivery is also polarized to cell protruding areas in an ECM-specific manner and contributes to FA growth and cell protrusion. (F–H) One-way ANOVA, Holm-Šídák’s multiple comparison test, data distribution was assumed to be normal, but this was not formally tested. **(F)***N* = 64 cells on collagen, 50 cells on FN, pooled from three independent experiments. **(G)** 57 WT cells and 52 ITGA5 KO cells, (H) 59 WT cells and 55 ITGA5 KO cells, pooled from three independent experiments. **(I)** Mann–Whitney test, *N* = 55 cells on FN, 66 cells on collagen, pooled from three independent experiments.

The endo/exocytic traffic of cell surface integrins controls FA dynamics, size, and distribution in cells (Ezratty et al., 2009; Moreno-Layseca et al., 2021; Sahgal et al., 2019; Nader et al., 2016); however, the role of integrin secretion remains to be explored. CD59, along with several other cargo proteins, undergo anterograde post-Golgi traffic to secretion hotspots adjacent to but discrete from FAs (Fourriere et al., 2019). We employed dual color TIRF imaging of RUSH-α5 and pmKate2-Paxillin in cells plated on FN to determine whether this is also the case for integrins. SPI revealed that RUSH-α5 is recruited to FAs and in their vicinity. When initiating a new adhesion, RUSH-α5 was initially localized to the most distal area (Area 1) of FAs (closest to the cell periphery), after which it gradually accumulated along the growing adhesion towards the cell center (Fig. 6, A and B; and Fig. S5 F). However, in an existing and elongated FA, RUSH-α5 was equally localized all along the FA (Fig. 6 C and Fig. S5 G).

To consider the effects of increased integrin delivery on adhesions, we employed a molecular clutch model previously developed to simulate mechanosensitive growth of adhesions (Elosegui-Artola et al., 2016). In this model, talin-mediated mechanosensing (through force-induced unfolding) leads to adhesion growth, modeled as an increase in integrin density (Fig. 6 D). To understand the effect of integrin delivery, we reasoned that an increased delivery would result in a higher availability of integrins to be incorporated into adhesions. Thus, we modeled integrin delivery by tuning the parameter that sets the increase in integrin density that occurs upon talin unfolding (*d*_*add*_). Running the model with a base set of parameters taken from previous work (Table S1), modifying only the *d*_*add*_ parameter, and running the simulation as a function of time, indicated that delivery of new receptors is predicted to increase adhesion growth (Fig. 6 E). In concordance with the model, release of RUSH-α5 significantly increased adhesion area on FN, with no significant effect on collagen (Fig. 6 F). Adhesion growth supported by RUSH-α5 release was also apparent in the ITGA5 KO cells (Fig. 6 G), indicating that an increased number of clutches translates to larger adhesions also when a new type of integrin heterodimer is introduced to the cell surface. The increase in adhesion size correlated with enhanced cell spreading in WT and ITGA5 KO cells transfected with RUSH-α5 (Fig. 6 H) and to increased cell dynamics with longer cell protrusions extended in cells plated on FN compared to collagen following biotin addition (Fig. 6 I; and Fig. S5, H and I). Taken together, these data indicate that newly synthesized integrin α5 is rapidly localized to FAs, contributing to adhesion growth towards the proximal end and facilitating cell protrusion in a spatially defined and ligand-dependent manner.

## Discussion

Our findings demonstrate that cell adhesion and polarized cell dynamics can be steered by targeted delivery of newly synthesized integrins to the plasma membrane, and that this can occur rapidly, in a localized manner, bypassing the Golgi complex (Fig. 6 J). The biosynthetic trafficking route of integrins has not been previously investigated spatiotemporally in cells or associated with dynamic regulation of cell morphology and integrin adhesions. Earlier biochemical labeling studies analyzed synthesis, folding and secretion of integrins but were not amendable for imaging of their delivery to the plasma membrane. Therefore, the contribution of newly synthesized integrin to cell dynamics has remained uninvestigated. Our findings place integrin delivery via the biosynthetic pathway on par with well-established pathways that control integrin dynamics on the plasma membrane, namely endocytosis and recycling.

Motor-clutch-based modeling has been employed to understand many fundamental aspects of cell dynamics in response to ECM ligands and matrix rigidity (Elosegui-Artola et al., 2016; Isomursu et al., 2022; Kechagia et al., 2019). Here, the model predicted time-dependent growth of adhesions in response to delivery of an increased number of integrins. These matched with our experimental data revealing increased adhesion growth and generation of cell protrusions in response to RUSH-α5 release from the ER. These data further underline that biosynthetic delivery of integrins is an important and thus far underestimated contributor to cell responses to ECM mechanics and composition.

Many open questions remain regarding the biological significance of integrin delivery via the biosynthetic route and the mechanisms governing this process. We find that GRASP65 is required for the rapid unconventional secretion of RUSH-α5. However, its full contribution to the spatial and temporal regulation of integrin unconventional secretion requires further investigation. The GRASP proteins are localized to the Golgi and are classically associated with Golgi stacking. However, they are also involved in stress-induced UPS (Giuliani et al., 2011). Our data imply that integrin UPS is adhesion-dependent and seems to be always linked with cell spreading. Therefore, it is tempting to speculate that local mechanical stress could be a signal for one pool of integrins to rapidly exit the ER and be delivered to the proximal plasma membrane. However, this remains to be investigated. While proteins can undergo N-glycosylation in the ER (Xiang et al., 2013), it is plausible that the glycans of the early secreted pool of integrins are not fully processed but rather remain in an immature, high-mannose state. N-glycan chains support integrin headpiece opening (activation) and increase integrin-ligand binding affinity (Li et al., 2017). However, high-mannose integrins are also functional in ECM engagement (Li et al., 2017), consistent with the early delivery of RUSH-α5 supporting cell protrusions and adhesion growth. The presence of high-mannose glycosylated integrins at the cell surface has been so far only demonstrated in cancer cells (Oh et al., 2022; Park et al., 2020). Moreover, we showed integrin delivery via unconventional secretion only in a cancer cell line model. Whether this is also relevant to normal cells or is specific to cancer cells remains to be determined.

Our data indicate that cells sense their underlying ECM and have the ability to deliver new integrins in a polarized manner in response to receptor-specific ECM ligands. This is an exciting observation that might be particularly relevant in the context of development, tissue patterning and in wound healing where directed cell migration is paramount (Kanchanawong and Calderwood, 2022). Thus far, we have explored this process and its regulation in the context of the FN-binding integrin α5β1. Existing data suggest that for all β1-integrins the β1-subunit is produced in excess and localizes to the ER where it homodimerizes with an α-subunit to become transported to the plasma membrane (Heino et al., 1989). Integrin α-subunits harbor distinct cytoplasmic domains, and only a subset have a putative GRASP PDZ-binding motif (De Franceschi et al., 2016). In the future it will be interesting to explore whether integrin UPS is applicable only to a subset of integrins and whether these are perhaps biologically differentially employed in processes requiring a rapid and dynamic cell response.

The field of cell-ECM adhesion is well-established and there is a wide consensus that endocytosis and recycling of integrins from and to the plasma membrane are essential regulators of cell dynamics, migration and invasion (Paul et al., 2015; Moreno-Layseca et al., 2019; Mana et al., 2020; Shafaq-Zadah et al., 2020; Warner et al., 2019). Dysregulation of many of these integrin trafficking regulators is also linked to cancer progression (Mana et al., 2020). We find here that delivery of fresh integrins, along the biosynthetic pathway, also operates to determine cell dynamics. Thus, mechanisms regulating integrin secretion are likely to be intertwined with established integrin trafficking pathways in previously unappreciated ways and most likely these mechanisms are relevant to human disease and operate alternately or even simultaneously endowing cells with greater plasticity to adapt to dynamic alterations in their extracellular environments.

### Limitations of the study

A large part of the results presented herein relies on the synchronized transport of a tagged integrin α5 using the RUSH assay. Therefore, it is important to note the limitations of the RUSH assay that might affect the interpretation of the results.

1. While tagging integrin α5 at its N-terminus (luminal domain) does not affect its heterodimerization with integrin β1 or ligand binding (demonstrated in Fig. 1), it is still possible that integrin α5 processing and/or its interaction with trafficking regulators are altered. Furthermore, there is the possibility that genetic manipulation to establish the hook may have slightly modified the integrin in a way that supports FA targeting.
2. The RUSH assay allows synchronized transport of a protein of interest (here integrin α5 or CD59) by retaining it in the ER. There, the protein is fully folded and ready to exit from the ER. We have demonstrated that the ER is not overloaded by the presence of the cargo as no unfolded protein response is detected (Boncompain et al., 2012). However, the sudden release of the cargo induced by the addition of biotin might lead to a slight overload of the secretory pathway. For this reason, in the study, a careful comparison of RUSH-α5 trafficking with another cargo (RUSH CD59) is conducted. Differential trafficking to FAs and sensitivity to ECM composition are observed. In theory, a synchronous arrival of high-mannose-containing cargos to the Golgi may overload the glycosylation machinery. However, we showed in our initial paper (Boncompain et al., 2012) that the glycosylation of RUSH cargos become endoglycosidase-resistant upon biotin addition suggesting that modification occurs normally in the Golgi. The RUSH system has been used extensively since and we are not aware of studies reporting glycosylation overflow using the RUSH assay.

## Materials and methods

### Cell culture

Chinese hamster ovary (CHO) cells (Esko et al., 1985) were a kind gift from the late Markku Salmivirta (University of Turku, Centre for Biotechnology, Turku, Finland) and grown in Ham’s F12 (15172529; Gibco) supplemented with 10% fetal calf serum (FCS, F7524; Sigma-Aldrich), and 1% L-glutamine (G7513; Sigma-Aldrich). U2OS cells (DSMZ; ACC 785) were grown in Dulbecco’s Modified Eagle’s medium (D5796; DMEM, Sigma-Aldrich) supplemented with 10% FCS, 1% L-glutamine and 1% penicillin/streptomycin (P0781; Sigma-Aldrich). Cells were routinely tested for mycoplasma contamination.

### CRIPSR/Cas9 ITGA5 KO clone generation

The U2OS ITGA5 KO clone was generated by CRISPR-Cas9 in the Finnish Genome Editing Unit (Sanger’s gRNA Library; Finnish Genome Editing Unit supported by HiLIFE and the Faculty of Medicine, University of Helsinki, and Biocenter Finland). U2OS cells were transfected with one gRNA and further grown as single cell clones. The KO efficiency was assessed by Western blot prior to TOPO cloning and Sanger sequencing to validate the KO in both alleles of the clone using the following primers: forward 5′-GGA​TTT​GGC​TTG​GGA​GGA​GAG​TAT​ATA​G-3′ and reverse 5′- CCA​GTC​CCT​CCC​TGA​ATT​TCA​C-3′. One clone from the procedure in which the KO was not efficient was used as a control WT clone.

### Plasmids

ERoxBFP was purchased from Addgene (68126) and pmKate2-paxillin from Evrogen (FP323). pEGFP-GRASP65 was a kind gift from Martin Lowe (University of Manchester, Manchester, UK). Briefly, the Lowe laboratory generated the plasmid by amplifying full-length GRASP65 by PCR and used in-frame cloning into the Xmal site of the mammalian expression vector pEGFP-N2 (Lane et al., 2002). Streptavidin-KDEL_SBP-mCherry-CD59 was generated by using the coding sequence corresponding to human CD59 (accession number CD59 (NP_000602) excluding its signal peptide and flanked with FseI and PacI restriction sites purchased as a synthetic gene from Eurofins Genomics. This fragment was inserted in Str-KDEL_ss-SBP-EGFP-CCR5 (Boncompain et al., 2019) using FseI and PacI restriction enzymes generating Str-KDEL_SBP-EGFP-CD59. Then, EGFP was replaced with mCherry (containing a silent mutation to remove SbfI internal site) taken from Str-KDEL-ss-mCherry-GPI (Boncompain et al., 2012) using SbfI and FseI restriction enzymes. The resulting plasmid Str-KDEL_SBP-mCherry-CD59 was verified by Sanger sequencing. Streptavidin-KDEL_SBP-EGFP-ITGA5 was generated by PCR amplification of human ITGA5 without its signal peptide using Integrin-α5-EGFP template from Patrick Caswell (University of Manchester, Manchester, UK) and the following PCR primers: forward 5′-AAT​TGG​CCG​GCC​GTT​CAA​CTT​AGA​CGC​GGA​GGC-3′ and reverse 5′-AAC​CTT​AAT​TAA​TCA​GGC​ATC​AGA​GGT​GGC​TGG-3′. The PCR fragment was then subcloned in the RUSH plasmid Streptavidin-KDEL_ss-SBP-EGFP (Boncompain et al., 2012) using FseI and PacI restriction enzymes. The EGFP was replaced by pHluorin to generate Streptavidin-KDEL_SBP-pHluorin-ITGA5 using FseI and SbfI restriction sites. The hook (streptavidin-KDEL) allows anchoring of the SBP-tagged reporter (integrin α5 and CD59) in the ER in the absence of biotin due to streptavidin–SBP interaction.

### Transfection

Plasmids of interest were transfected using Lipofectamine 3000, Lipofectamine 2000 (11668019 and L3000001; Thermo Fisher Scientific, respectively), or jetPRIME (101000027; Polyplus transfection) according to the manufacturers’ instructions. Protein downregulation was carried out with Lipofectamine siRNA Max or Lipofectamine 3000 (13778075 and L3000001; Thermo Fisher Scientific, respectively) according to manufacturer’s instructions. The siRNA used as control (siCTRL) was Allstars negative control siRNA (1027281; Qiagen). GRASP65 and GRASP55 were downregulated with Flexitube siRNAs (GS64689 and GS26003; Qiagen respectively) or custom ordered siRNA oligonucleotides (GRASP65 target sequence: AAG-GCA-CUA-CUG-AAA-GCC-AAU and GRASP55 target sequence: AAC-UGU-CGA-GAA-GUG-AUU-AUU; Qiagen).

### RUSH-α5 transfection and release

Cells grown to 25% confluence were used for transfection. For a 6 cm dish 1 × 10^5^ cells were transfected with 10 µg RUSH-α5 using Lipofectamine 3000. The cells were from this point grown in medium containing 1–2.5 µg/ml streptavidin (S4762; Sigma-Aldrich) to block biotin in the media and transfection reagents. The day after transfection, cells were used for experiments, either directly by releasing the RUSH or detaching the cells with trypsin and seeding the cells beforehand on appropriate surfaces for imaging experiments as described below. The release of the RUSH-α5 or RUSH-CD59 from the ER-hook was induced by replacing the medium with biotin-supplemented medium (3 mM of D-biotin; B4501; Sigma-Aldrich) for the indicated times.

### Immunoprecipitations and immunoblotting

#### GFP pulldown

CHO or U2OS cells expressing GFP-tagged (RUSH constructs) proteins (three 10 cm dishes per condition or one 10 cm dish, 80% confluence, for GFP-control construct, due to differences in expression efficiency) were washed with cold phosphate buffered saline (D8537-500 Ml; PBS Sigma-Aldrich), harvested in PBS and pelleted. The cell pellet was resuspended in 200 μl of IP-lysis buffer (40 mM Hepes-NaOH, 75 mM NaCl, 2 mM EDTA [E4884-100G; Sigma-Aldrich], 1% NP-40 [13434269; Thermo Fisher Scientific], protease and phosphatase inhibitors [0 505 648 9001 and 0 490 683 7001; Roche]) and incubated at 4°C for 30 min, followed by centrifugation (10,000 *g* for 10 min, 4°C). 20 μl of the supernatant was kept aside as the lysate control. The remainder of the supernatant was incubated with GFP-Trap beads (gtak-20; ChromoTek), for 55 min at 4°C.

#### AvFc lectibody pulldown

Two 10 cm dishes containing 80% confluent U2OS cells expressing RUSH-α5 were incubated with 3 mM biotin at 37°C for 1 h and then with 5 µg AvFc lectibody (Oh et al., 2022) in PBS on ice for 1 h. The cells were washed three times with PBS before addition of lysis buffer (1.5% Octylglycoside, 1% NP-40, 0.5% bovine serum albumin (BSA, 1 mM EDTA, protease and phosphatase inhibitors). The lysates were spun down and the supernatant was collected. 20 μl of the supernatant was kept aside as the lysate control. 25 μl of protein G beads (SureBeads Protein G Magnetic Beads; BioRad) were added to the remaining lysates and incubated for 2 h at 4°C.

#### GST pulldown

Pulldown with N-terminally biotinylated integrin α5 tail peptides (GenScript) was performed as follows: biotinylated peptides were incubated with streptavidin-conjugated Dynabeads (65001; Thermo Fisher Scientific) for 30 min at room temperature followed by a 2 h incubation with supernatant (prepared in the same way as described above for GFP-immunoprecipitated samples) from EGFP-GRASP65-overexpressing CHO cells or with recombinant GST-GRASP65 or GST alone (GenScript), at 4°C.

#### Immunoblotting

Immunoprecipitated complexes were washed three times with wash-buffer (20 mM Tris–HCl pH 7.5, 150 mM NaCl, 1% NP-40) or RIPA buffer (lectibody IP) (10 mM Tris–HCl pH8, 1 mM EDTA, 0.5 mM EGTA, 1% Triton X-100, 0.1% SodiumDeoxcholate, 0.1% SDS, 140 mM NaCl, protease and phosphatase inhibitors) and denatured for 10 min at 95°C in reducing Laemmli buffer before SDS-PAGE analysis under denaturing conditions (4–20% Mini-PROTEAN TGX Gels, #561096; Bio-Rad Laboratories). The proteins were then transferred to nitrocellulose membranes (#1704158; Bio-Rad Laboratories) before blocking with blocking buffer (#37538; StartingBlock blocking; Thermo Fisher Scientific) and PBS (1:1 ratio). The membranes were incubated with primary antibodies diluted in blocking buffer overnight at 4°C. Following this step, membranes were washed three times with TBST and incubated with fluorophore-conjugated secondary antibodies (LI-COR) diluted (1:10,000) in blocking buffer at room temperature for 1 h. Membranes were scanned using BioRad ChemiDoc MP Gel Analyzer, an infrared imaging system (Odyssey; LI-COR Biosciences) or Azure Sapphire RGBNIR Biomolecular Imager. Image J was used for further analysis of acquired images and protein band intensities. The relative fraction of mature to immature integrin β1 interacting with RUSH-α5 was quantified by dividing the integrin β1 intensity of the upper band (mature integrin β1) by the intensity of the lower band (immature integrin β1) from the GFP pulldown blot. Primary antibodies used: Mouse anti-CD29 (integrin β1) (610468; BD Biosciences), rabbit anti-GRASP55 (HPA035274; Sigma-Aldrich), rabbit anti-GRASP65 (HPA056283; Sigma-Aldrich), rabbit anti-GFP (ab290; abcam), mouse anti-GAPDH (5G4MaB6C5; Bioz), rabbit anti-GST tag (A-5800; Invitrogen). Secondary antibodies used: IRDye 800CW Donkey anti-mouse IgG (926-32212; LICOR), IRDye 800CW Donkey anti-rabbit IgG (926-32213; LICOR), IRDye 680LT Donkey anti-Mouse IgG (926-68022; LICOR), and IRDye 680LT Donkey anti-Rabbit IgG (926-68023; LICOR), diluted 1:10,000 in odyssey blocking buffer (927-40000; LICOR).

### ELISA

Wells of a Nunc Maxisorp 96-well plate (Sigma-Aldrich) were first incubated with GST or GST-GRASP65 (0-1-2-5 and 10 µg in 50 μl) overnight at 4°C. The wells were then washed with HMN-Tween 0.05% (HMN-T, HMN buffer: 20 mM Hepes, 100 mM NaCl, 5 mM MgCl2), blocked with 1% BSA in HMN-T for 2 h on ice and washed with HMN-T. 0.2 µg of integrin β1 or integrin α5 tail peptides were incubated in the wells for 2 h at room temperature. After two washes with HMN-T, HRP-streptavidin was added for 1 h at room temperature and the wells were washed again twice. 100 μl of TMB ELISA reagent was added and incubated for 10 min. The reaction was stopped with H_2_SO_4_ 2M and absorption at 450 nm was measured with a plate reader.

### Flow cytometry

Cells were detached on ice, before or after biotin addition in the case of RUSH-α5 transfected cells, with enzyme-free cell dissociation buffer (13150016; Gibco). Pelleted cells were incubated with anti-GFP-AF647 antibody (1:150, Alexa Fluor 647 Mouse Anti-GFP Clone 1A12-6-18, 565197; BD Biosciences), rabbit anti-ITGA5 (1:100, clone EPR7854; ab150361; Abcam) or HiLyte Fluor 647-labeled PFL (0.5 µM) (Sato et al., 2012), in Tyrodes Buffer (10 mM HEPES-NaOH at pH 7.5, 137 mM NaCl, 2.68 mM KCl, 1.7 mM MgCl_2_, 11.9 mM NaHCO_3_, 5 mM glucose, 0.1% BSA) for 40 min on ice. For PFL staining, cells were blocked with 5 µM unlabeled PFL before biotin addition. Cells were washed twice with Tyrodes Buffer. In the case of anti-ITGA5 staining, cells were then incubated with a donkey anti-Rabbit-AF647 (1:400; A31573; Invitrogen) for 30 min on ice and washed twice. Cells were fixed for 10 min with 2% PFA, resuspended in PBS and analyzed with LSRFortessa (BD Biosciences). Data analysis was performed with FlowJo software version 5. To quantify cell surface RUSH-α5 levels, the geometric mean of the anti-GFP AF647 antibody or PFL647 signal (surface labeling) was divided by the total GFP signal for each time point, and the value at T0 was subtracted from all time points.

### Live-cell imaging

Cells were plated in imaging media (1:1 ratio of DMEM [D5796; Sigma-Aldrich] and FluoroBrite DMEM Media [A189670; Thermo Fisher Scientific], supplemented with 20 mM HEPES [15630080; Thermo Fisher Scientific], 1 µg/ml streptavidin [S4762; Sigma-Aldrich] 10% fetal calf serum (FCS, F7524; Sigma-Aldrich), 1% L-glutamine (G7513; Sigma-Aldrich), and 1% penicillin/streptomycin [P0781; Sigma-Aldrich] and allowed to spread for 2–4 h before imaging on FN-coated [341631; Merck-Millipore] or collagen-coated [catalog number 08-115; Merck-Millipore] [10 µg/ml]) coverslips. Additional coating of 2.5 µg/ml Alpaca anti-GFP VHH nanobody (gt-250; Chromotek) was used in the indicated experiments. Time-lapse imaging was performed at 37°C using a Spinning-disk confocal 3i (Intelligent Imaging Innovations, 3i Inc.) Marianas Spinning disk confocal microscope with a Yokogawa CSU-W1 scanner and back illuminated 10 MHz EMCCD camera (Photometrics Evolve) using a 63x/1.4 oil objective. TIRF imaging was carried out using a DeltaVision OMX with a 60x/1.49 Olympus APO N TIRF objective. Conventional protein secretion was blocked (in indicated experiments) by incubating the cells with 10 µM Golgicide A (G0923; Sigma-Aldrich) 30 min prior to imaging. The release of the RUSH cargos was induced by removing the streptavidin supplemented media and addition of biotin-supplemented imaging media (3 mM of D-biotin, B4501; Sigma-Aldrich), by using a magnetic imaging chamber with L-shape tubing (CM-B25-1PB; Live Cell Instrument CO., LTD) during the live-cell imaging experiments.

### Image analyses

As the intensity of RUSH-α5 varied from cell to cell based on transfection efficiency, relative RUSH-α5 recruitment was measured by normalizing the intensity at the indicated time point to the intensity before release (0 min) in the same measured region. Due to the low exposure time used for image acquisition of pmKate2-Paxillin (to reduce phototoxicity), de-noising of paxillin adhesions was carried out using the deep learning CARE2D network (Weigert et al., 2018) in the ZeroCostDL4Mic platform (von Chamier et al., 2021), where 200 images were used to train the model prior to analysis. Paxillin adhesions were then segmented when needed. Images of paxillin adhesions were made binary and adhesions larger than 0.6 µm^2^ were segmented and analyzed with the Analyze Particles tool in ImageJ (Schindelin et al., 2012). The segmented adhesions were saved as regions of interest (ROIs) in the ROI manager and used to measure the intensity of RUSH-α5 within the paxillin adhesions. Spatiotemporal track maps of cells were generated based on the RUSH-α5 signal using the QuimP plugin (Baniukiewicz et al., 2018) in ImageJ. Localization of RUSH-α5 to adhesions was studied by drawing a ROI around the whole adhesion area based on the paxillin signal and then dividing the ROI into 4 areas of equal size. RUSH-α5 intensity in the four areas relative to signal intensity in the respective area 2.5 min prior to RUSH-α5 appearance in adhesions (determined from time-lapse imaging) was measured. Adhesions close to the cell edge and with a minimum lifetime of 15 min were analyzed and changes of RUSH-α5 intensity were plotted over time in the indicated areas ranging from distal to proximal to the cell body. Detection of exocytosis events from the TIRF RUSH-α5-pHluorin imaging was performed by dividing each frame by the previous with the ImageJ image calculator function. Exocytosis spots were then segmented by manual thresholding. The last frame was used to segment FAs using trainable WEKA segmentation (Arganda-Carreras et al., 2017). The nearest distance between exocytosis events or randomly generated spots and focal adhesions was measured with the Distance Analysis (DiAna) Fiji plugin (Gilles et al., 2017).

### Immunofluorescence and image acquisition of fixed samples

Cells were plated on Ibidi 35 mm µ-dishes (80136) coated with 10 µg/ml collagen I (catalog number 08-115; Merck-Millipore) or FN (341631; Merck-Millipore). Samples were fixed for 10 min with 4% PFA followed by permeabilization for 10 min with 0.1% Triton X-100 in PBS. For ER staining with anti-PDI, samples were fixed in warm PEM buffer (8 mM PIPES, 5 mM EGTA and 2 mM MgCl_2_) containing 0.5% glutaraldehyde and 0.25% Triton X-100 for 10 min at 37°C. Glutaraldehyde was then quenched with 0.1% NaBH_4_ for 7 min. To block nonspecific binding of antibodies, cells were incubated in 10 % horse serum (16050–122; HRS; Gibco) for 1 h or in 1 M Glycine for 20 min at room temperature. Primary and secondary antibodies were diluted in 10% HRS and incubated for 1 h at room temperature. Primary antibodies used: mouse anti-integrin α5 (clone SNAKA51) (MABT201, 1:500; Millipore), mouse anti-GM130 (clone 35/GM130; BD Biosciences, 1:1,000), and mouse anti-PDI (clone 1D3; Enzo, 1:100). Secondary antibodies used were Alexa Fluor 555 anti-mouse (A32727; Thermo Fisher Scientific, 1:400), Alexa Fluor 568 anti-mouse (1:400; A10037; Thermo Fisher Scientific). F-actin was stained with Phalloidin-Atto 647N (1:400, 65906; Sigma-Aldrich), incubated together with secondary antibodies. Nuclei were stained with DAPI (1:3,000; D1306; Life Technologies) for 10 min at room temperature after secondary antibody incubation. Samples were imaged using either A) 3i (Intelligent Imaging Innovations, 3i Inc.) Marianas Spinning disk confocal microscope with a Yokogawa CSU-W1 scanner and Hamamatsu sCMOS Orca Flash 4.0 camera (Hamamatsu Photonics K.K.) or back illuminated 10 MHz EMCCD camera (Photometrics Evolve) using 63×/1.4 oil or 40×/1.1 water objectives; B) Zeiss LSM780 laser scanning confocal microscope using a 40×/1.2 water Zeiss C-Apochromat objective; or C) LSM880 laser scanning confocal microscope with AiryScan using a 63×/1.4 oil Zeiss C Plan-Apochromat objective for high resolution imaging.

### Micropatterns

Micropatterns were produced on glass coverslips as described in Azioune et al. (2009). Briefly, glass coverslips were washed with ethanol and exposed to deep UV for 5 min followed by 1 h incubation with 0.1 mg/ml PEG-g-PLL (Surface Solutions, Zurich) in 10 mM HEPES pH 7.4 at room temperature. Coated coverslips were washed twice with PBS, once with H_2_O, and left to dry. The PEG-g-PLL coated coverslip was then placed on a photomask and exposed to deep UV for 6 min. Micropatterned coverslips were then coated with FN (341631; Merck-Millipore) or collagen (catalog number 08-115; Merck-Millipore) (10 µg/ml), together with 2.5 µg/ml Alpaca anti-GFP VHH nanobody (gt-250; Chromotek) to trap the released RUSH-α5 via its EGFP domain, and 5 µg/ml BSA Alexa Fluor 647 conjugate (A34785; Thermo Fisher Scientific) to visualize the micropattern. Cells were seeded in culture medium with FN-depleted serum and allowed to spread on micropatterns for a minimum of 2 h. For experiments with dual-coated micropatterns, PLL(20)-g[3.5]-PEG(2)/PEG(3.4)-biotin (50%) (SuSoS) was used in a recently developed method (Isomursu et al., 2024) allowing coating of the non-micropatterned areas: the micropatterned areas were first coated with 50 µg/ml FN and 5 µg/ml BSA Alexa Fluor 647 conjugate followed by 30 min blocking with 3% BSA, then the non-micropatterned areas were coated with 10 µg/ml GFOGER (Auspep) conjugated to streptavidin using the FastLink Streptavidin Labeling Kit (KA1556; Abnova) according to manufacturer’s instruction.

### Constructing complete atomistic and coarse-grained models

We constructed simulation models that match the protein complexes studied in experiments as accurately as possible. To this end, we built both an atomistic and a coarse-grained Martini 3 (Souza et al., 2021) model of the bound integrin construct as described here and in the next paragraph. For the FN and antibody-bound integrin structure, we used the PDB id 7NWL, with the bound antibody removed. In this structure, the transmembrane and intracellular domains of the integrin alpha and beta are not included. As the starting structure for EGFP, we used the PDB id 2Y0G, where we did not include the three chromatic residues (TYG) in 2Y0G as they are non-standard. To speed up the construction of the complete protein complex, these atomistic structures were individually coarse-grained to the Martini 3 representation with elastic networks and then put together in a single box ([Periole et al., 2009]; https://github.com/marrink-lab/vermouth-martinize). The box was solvated with default Martini 3 water at 150 mM NaCl. In this coarse-grained representation, the EGFP C-terminus was pulled toward the integrin alpha N-terminus (constant rate at 1 nm ns^−1^ with a harmonic force constant of 1,000 kJ mol^−1^ nm^−2^) to reach a structure where the termini were <1 nm apart. When this state was achieved, the coarse-grained structure (the FN-integrin-EGFP complex) was backmapped to an atomistic representation by aligning the atomistic structures to the pulled coarse-grained system using the C-alpha backbone beads for reference. Finally, as the last few residues at the C-terminus of the EGFP (2Y0G) structure were missing (LGMDELYK), they were added together with the linker using the Modeller loop protocol (Webb and Sali, 2016), i.e., the extended EGFP C-terminus was connected to the N-terminus of the integrin alpha subunit of 7NWL. This final atomistic model contained all sugars and bound ions from the crystal structures and was used as the starting structure in atomistic molecular dynamics simulations. Further, this final atomistic structure of the complete complex was coarse-grained to the Martini 3 representation, which was used as the starting structure in the construction of the complete coarse-grained simulation model (see the next paragraph).

### Constructing the production coarse-grained model

For the Martini 3, coarse-grained production simulations the sugars and manganese cations in the 7NWL structure were not taken into consideration. To maintain the folded structure, an elastic network was added using Martinize2 based on the ElNeDyn protocol (Periole et al., 2009). Both intra- and intermolecular contacts were stabilized by the elastic network, but no harmonic bonds were added between EGFP and integrins/FN. The elastic bonds in the linker residues were removed completely. The complex was solvated using insane (INSert membrANE) in default Martini 3 water with 150 mM NaCl (Wassenaar et al., 2015) with a minimum periodic distance of 4 nm.

### Simulations of the production coarse-grained models

To run the coarse-grained simulations, we used the GROMACS 2021.2 package (Abraham et al., 2015). For energy minimization, the steepest descent algorithm was used, and during equilibration the default Martini settings were employed, making use of a 1 fs time step up to the point that numerical stability was achieved (de Jong et al., 2016). The Verlet cutoff scheme was used with a 1.1 nm cutoff for both the Coulombic (reaction-field) and van der Waals interactions. We used v-rescale for the thermostat at 300 K, coupling the protein and solvent in separate groups. Pressure coupling was initially performed using the Berendsen barostat (Berendsen et al., 1984) for isotropic systems at 1 atm. The production runs made use of a 20 fs time step, where the pressure coupling was switched to Parrinello-Rahman (Parrinello and Rahman, 1981). For the pulling simulations, the pull code as implemented in GROMACS 2021.2 was used. The umbrella pulling method was employed to pull EGFP along a vector joining the center of mass (COM) of EGFP towards the FN-binding site. For each pulling simulation, a rate of 0.1 nm ns^-1^ was used with a harmonic force constant of 1,000 kJ mol^−1^ nm^−2^. The videos were made with the VMD movie maker plugin (Humphrey et al., 1996). The production runs spanned 80 ns for the binding site pulling and 3,200 ns for the subsequent (non-biased) relaxation.

### Simulations of the atomistic models

To run the atomistic simulations, we used the GROMACS 2021.2 package (Abraham et al., 2015). The protein was solvated in the presence of 150 mM of sodium chloride at 310 K temperature with a pressure of 1 atm. The LINCS algorithm (Hess, 2008) was used to constrain the bond lengths in the system during simulations. The CHARMM36m force field (Huang et al., 2017) was used to derive the parameters for the protein and the ions. The CHARMM water model (MacKerell et al., 1998) was used to obtain parameters for the water molecules used to solvate the protein. The particle mesh Ewald technique (Darden et al., 1993) was used to calculate electrostatic interactions within the simulation system with a real-space cutoff of 1.2 nm. The protein structure was first energy-minimized and then subjected to a 100 ns equilibration. 10 independent simulations were then performed for generating the production runs. A time step of 4 fs was used for the simulations using the hydrogen mass partitioning method (Hopkins et al., 2015). For the pulling simulations, the pull code as implemented in GROMACS 2021.2 was used. The umbrella pulling method was employed to pull the EGFP along a vector joining the COM of EGFP towards the FN-binding site. For each pulling simulation, a rate of 0.1 nm/ns was used. The videos were made with the VMD Movie maker plugin (Humphrey et al., 1996).

### Computational clutch model

The clutch model used considers how force transmitted from myosin motors to the substrate is applied to talin molecules and integrin-substrate bonds. Integrins bind and unbind from the substrate through binding rate *k*_*on*_ and unbinding rate *k*_*off*_, and talin folds and unfolds with folding and unfolding rates *k*_*fold*_ and *k*_*unfold*_. *K*_*off*_, *k*_*fold*_, and *k*_*unfold*_ depend on force as previously described experimentally. Binding sites on the substrate are modeled explicitly, whereas integrins are modeled implicitly via a given integrin density *d*_*int*_. Each time that talin unfolds an adhesion reinforcement event is assumed to happen, which is modeled as an increase in integrin density *d*_*add*_. Model code and all parameters were taken from previous work (Elosegui-Artola et al., 2016). The only differences were the following.

• Our previous work considered that integrin density could both increase (when talin unfolds) and decrease (when integrins unbind from the substrate without talin unfolding). Here, we are only modeling adhesion growth, so we only consider growth.
• We set an upper limit to integrin density (three times the initial value), to consider that adhesions only grow to a maximum size.
• We decreased the parameter *d*_*add*_ to match the timescale of adhesion growth (to 0.01 or 0.005 integrins/μm^2^).

### Statistical analysis

GraphPad Prism software was used and the names and/or numbers of individual statistical tests, samples and data points are indicated in figure legends. Unless otherwise noted, all results are representative of three independent experiments and P values <0.05 are shown in graphs.

### Online supplemental material

Fig. S1 shows the RUSH system applied to integrin α5. Fig. S2 shows RUSH-α5 recruitment to adhesions is ligand-dependent. Fig. S3 shows RUSH-α5 delivery and localization following release. Fig. S4 shows early release of RUSH-α5 is adhesion dependent and polarized recruitment to protrusion is supported by endogenous integrin α5. Fig. S5 shows early release of RUSH-α5 is sensitive to GRASP silencing. Video 1 shows a rotating model of RUSH-α5–integrin-β1 heterodimer bound to FN. Video 2 A shows for the coarse-grained model, the pulling of EGFP towards the FN-binding site. Video 2 B shows for the coarse-grained simulation model, the spontaneous relaxation of the final pulled state of Video 2 A. Video 3 A shows fully atomistic molecular dynamics simulation of the EGFP attached to the α-subunit of the integrin molecule without applying forces. Video 3 B shows fully atomistic steered molecular dynamics simulation of the EGFP attached to the α-subunit of the integrin molecule with a pulling force of 25 kJ/mol/nm^2^. Video 3 C shows fully atomistic steered molecular dynamics simulation of the EGFP attached to the α-subunit of the integrin molecule with a pulling force of 50 kJ/mol/nm^2^. Video 4 shows time lapse imaging of RUSH-α5–expressing U2OS cell on FN. Video 5 shows time lapse imaging of U2OS cells co-expressing RUSH-α5 and RUSH-CD59 on FN or collagen. Video 6 shows time lapse TIRF imaging of U2OS expressing RUSH-α5-pHluorin on FN. Video 7 shows time lapse imaging of U2OS expressing RUSH-α5 on FN and anti-GFP-coated micropattern lines. Video 8 shows time lapse imaging of U2OS expressing RUSH-α5 on collagen and anti-GFP-coated micropattern lines. Video 9 shows time lapse imaging of U2OS cells co-expressing RUSH-α5 and an ER marker on FN. Video 10 shows time lapse TIRF imaging of U2OS cells expressing RUSH-α5 on FN. Table S1 shows molecular clutch model parameters. SourceDataF5 shows uncropped and unprocessed blots for Fig. 5.

## Supplementary Material

Table S1 Model parameters.

SourceData F5 is the source file for Fig. 5.

SourceData FS1 is the source file for Fig. S1.

SourceData FS2 is the source file for Fig. S2.

SourceData FS3 is the source file for Fig. S3.

SourceData FS4 is the source file for Fig. S4.

SourceData FS5 is the source file for Fig. S5.

## Data Availability

The data supporting the findings of this study are available within the article and from the authors on reasonable request.
