# Supplementary material for: Regulation of cell dynamics by rapid integrin transport through the biosynthetic pathway
Source: J Cell Biol. 2025 Dec 2;225(2):e202508155. doi: 10.1083/jcb.202508155 (PMC12671483; doi:10.1083/jcb.202508155)
Supplement: Table S1 — Model parameters. [file jcb_202508155_tables1.docx]

**Supplementary Table 1.** Model parameters. Note that all parameters are taken from (Elosegui-Artola, A. et al. 2016) except for *d_add_* and *d_max_*.

| **Parameter** | **meaning** | **Value** | **Origin** |
| --- | --- | --- | --- |
| *n_f_* | Number of fibronectin ligands | 1200 | Elosegui-Artola, A. et al. 2016 |
| *k_ont_* | True binding rate | 2.11x10^-4^ um^2^/s | Elosegui-Artola, A. et al. 2016 |
| *n_m_* | Number of myosin motors | 800 | Elosegui-Artola, A. et al. 2016 |
| *d_int_* | Initial integrin density on the membrane | 300 /μm^2^ | Elosegui-Artola, A. et al. 2016 |
| *d_add_* | Integrins added after each reinforcement event | 0.005 /μm^2^ (low delivery)  0.01 /μm^2^  (high delivery) | adjusted |
| *d_max_* | Maximum possible integrin density | 900 /μm^2^ | adjusted |
| *FR* | Fraction of force experienced by talin | 0.073 | Elosegui-Artola, A. et al. 2016 |
| *F_m_* | Myosin motor stall force | *2 pN* | Elosegui-Artola, A. et al. 2016 |
| *v_u_* | Unloaded myosin motor velocity | 110 nm/s | Elosegui-Artola, A. et al. 2016 |
| *K_off_* | Integrin unbinding rate | Catch bond | Elosegui-Artola, A. et al. 2016 |
| *K_fold_* | Talin unfolding rate | Slip bond | Elosegui-Artola, A. et al. 2016 |
| *K_unfold_* | Talin refolding rate | Slip bond | Elosegui-Artola, A. et al. 2016 |
| *k_c_* | Clutch spring constant | 1 nN/nm | Elosegui-Artola, A. et al. 2016 |
| *k_sub_* | Substrate spring constant | 0.1 N/m | Highest value used in Elosegui-Artola, A. et al. 2016 (corresponding to very stiff substrates such as glass) |
