# Supplementary figures and images for: Regulation of cell dynamics by rapid integrin transport through the biosynthetic pathway
Source: J Cell Biol. 2025 Dec 2;225(2):e202508155. doi: 10.1083/jcb.202508155 (PMC12671483; doi:10.1083/jcb.202508155)

Figure 5c

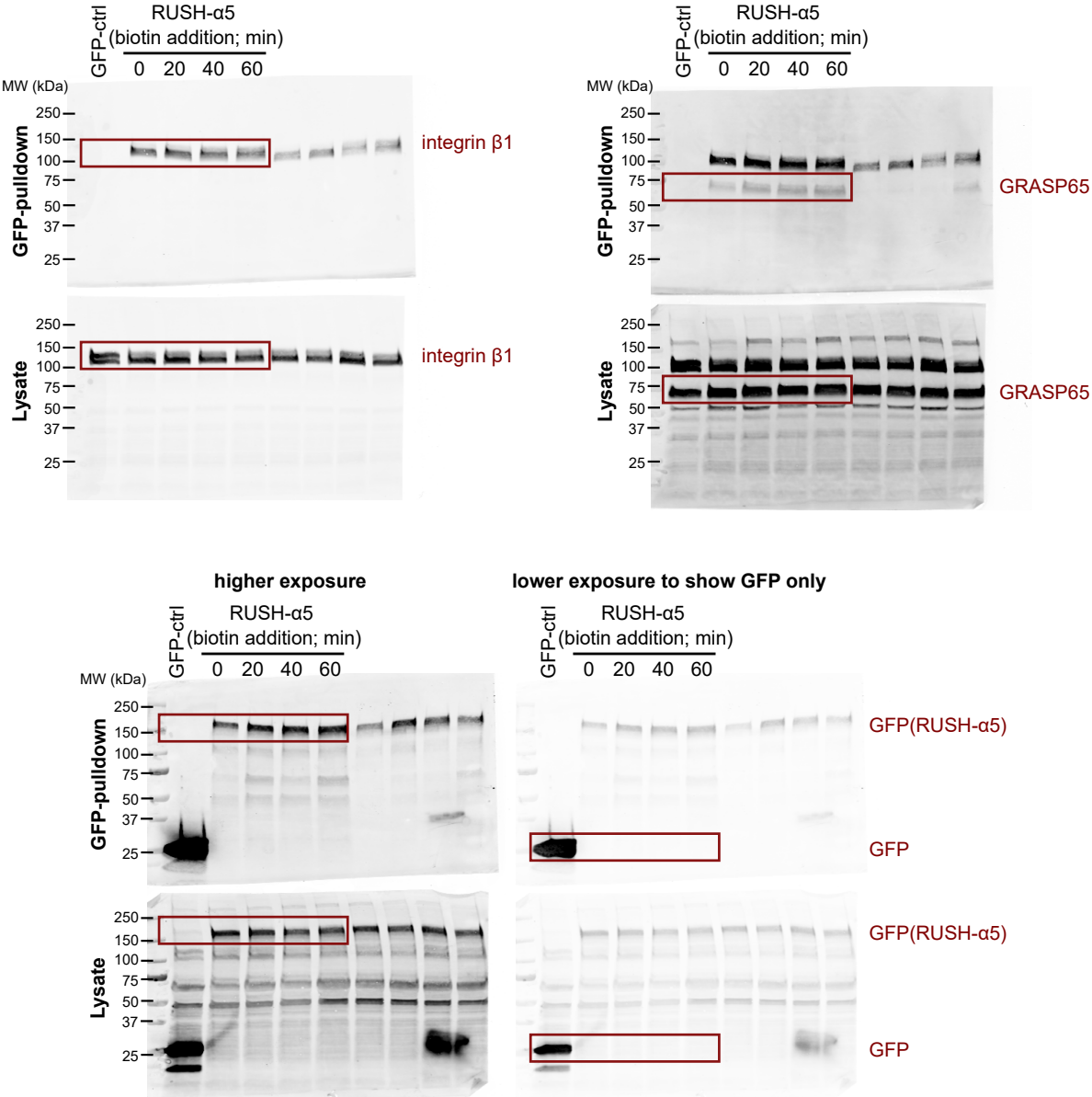

Figure 5e

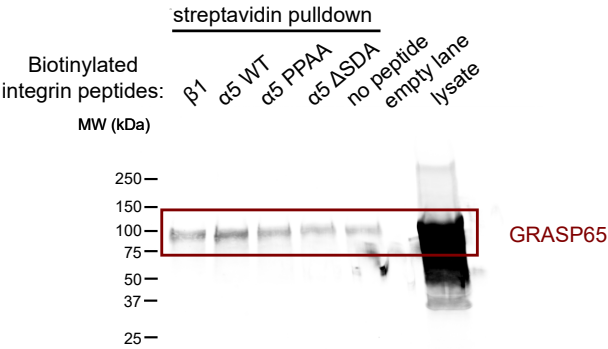

Supplement: SourceData F5 — is the source file for Fig. 5. [file jcb_202508155_sourcedataf5.pdf]

Figure S1d

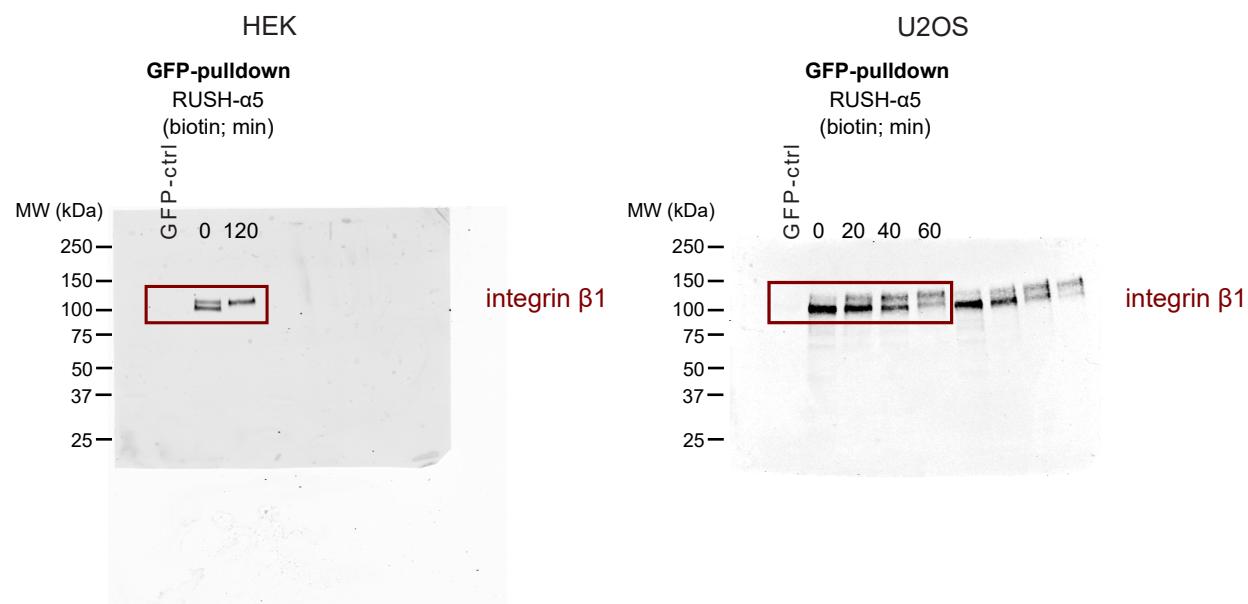

Supplement: SourceData FS1 — is the source file for Fig. S1. [file jcb_202508155_sourcedatafs1.pdf]

Figure S3d

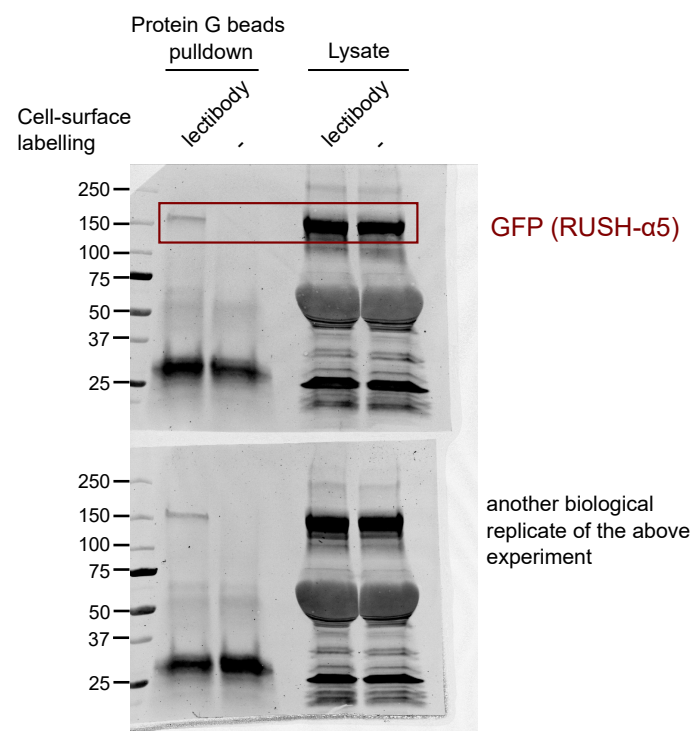

Supplement: SourceData FS3 — is the source file for Fig. S3. [file jcb_202508155_sourcedatafs3.pdf]

Figure S4a

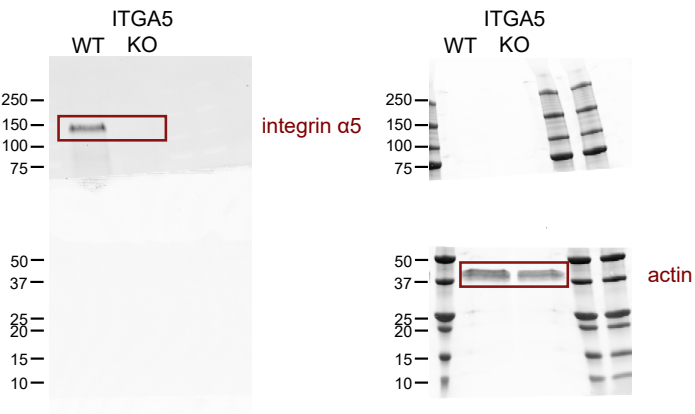

Supplement: SourceData FS4 — is the source file for Fig. S4. [file jcb_202508155_sourcedatafs4.pdf]

Figure S5a

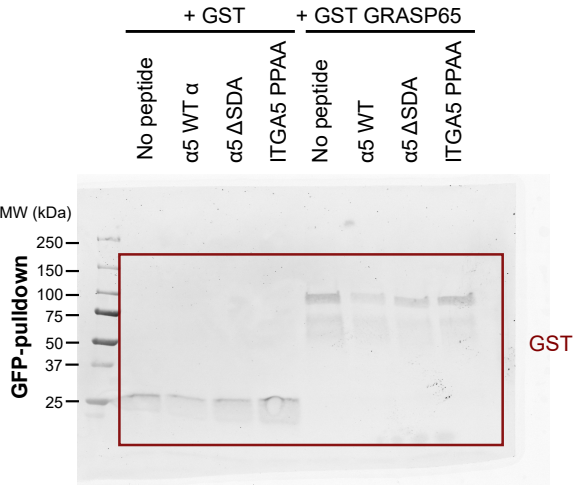

Figure S5c

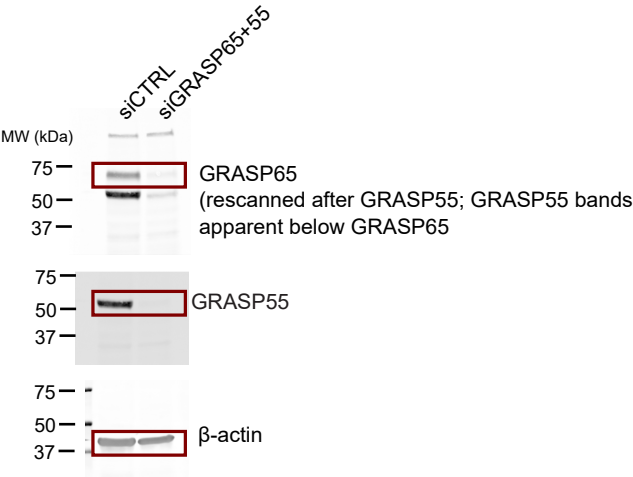

Supplement: SourceData FS5 — is the source file for Fig. S5. [file jcb_202508155_sourcedatafs5.pdf]
