# Supplementary material for: Regulation of cell dynamics by rapid integrin transport through the biosynthetic pathway
Source: J Cell Biol. 2025 Dec 2;225(2):e202508155. doi: 10.1083/jcb.202508155 (PMC12671483; doi:10.1083/jcb.202508155)
Supplement: SourceData FS2 — is the source file for Fig. S2. [file jcb_202508155_sourcedatafs2.pdf]

Figure S2a

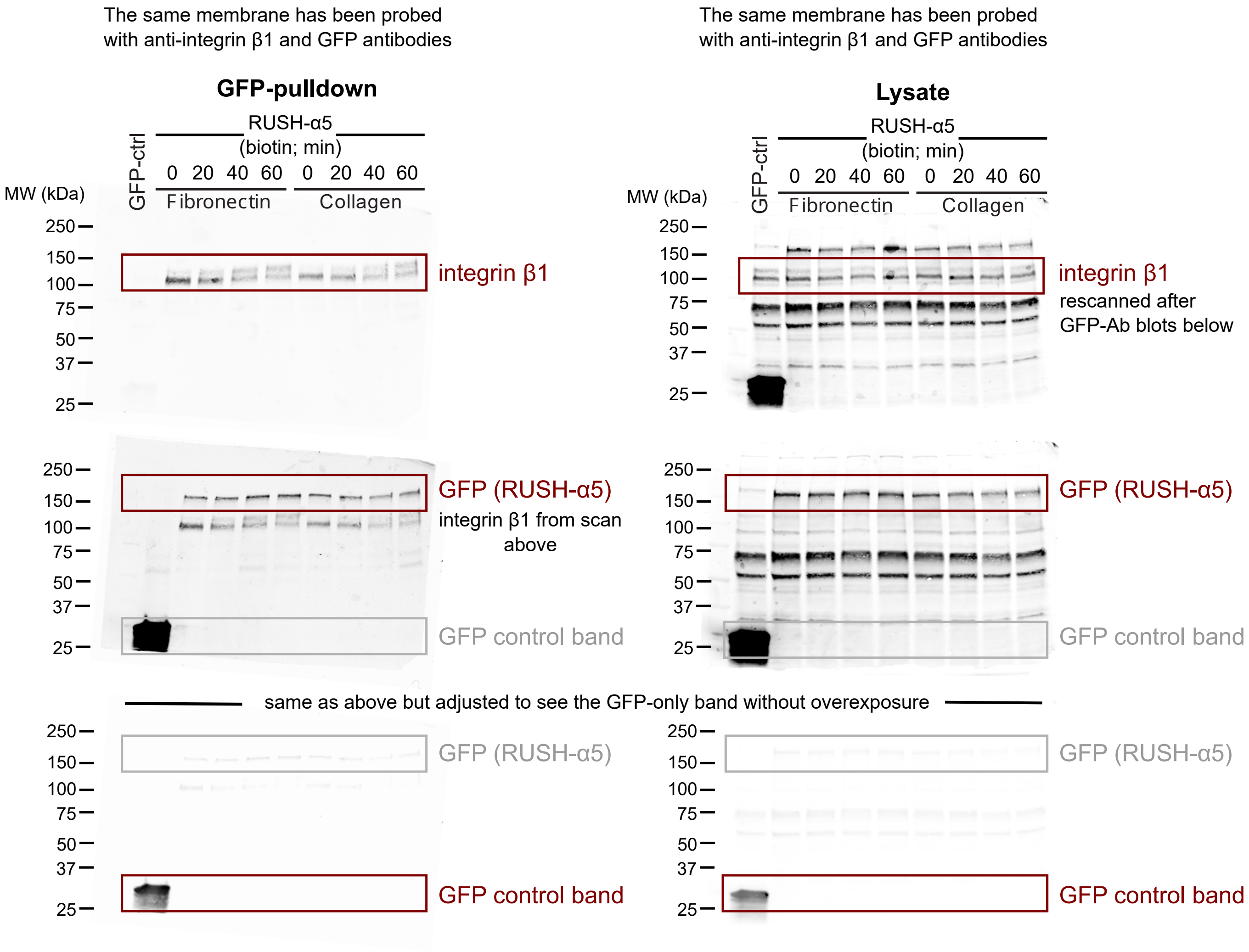

The same membrane has been probed with anti-integrin β1 and GFP antibodies
GFP-pulldown
RUSH-α5
(biotin; min)
GFP-ctrl
0 20 40 60 0 20 40 60
Fibronectin
Collagen
MW (kDa)
250
150
100
75
50
37
25
integrin β1
GFP (RUSH-α5)
integrin β1 from scan above
GFP control band
The same membrane has been probed with anti-integrin β1 and GFP antibodies
Lysate
integrin β1
rescanned after GFP-Ab blots below
GFP (RUSH-α5)
GFP control band
same as above but adjusted to see the GFP-only band without overexposure
GFP (RUSH-α5)
GFP control band
